# A PCR Based Protocol for Detecting Indel Mutations Induced by TALENs and CRISPR/Cas9 in Zebrafish

**DOI:** 10.1371/journal.pone.0098282

**Published:** 2014-06-05

**Authors:** Chuan Yu, Yaguang Zhang, Shaohua Yao, Yuquan Wei

**Affiliations:** State Key Laboratory of Biotherapy and Cancer Center, West China Hospital and College of Life Science, Sichuan University, Chengdu, Sichuan, People’s Republic of China; University College London, United Kingdom

## Abstract

Genome editing techniques such as the zinc-finger nucleases (ZFNs), transcription activator-like effecter nucleases (TALENs) and clustered regularly interspaced short palindromic repeats (CRISPR)/CRISPR-associated (Cas) system Cas9 can induce efficient DNA double strand breaks (DSBs) at the target genomic sequence and result in indel mutations by the error-prone non-homologous end joining (NHEJ) DNA repair system. Several methods including sequence specific endonuclease assay, T7E1 assay and high resolution melting curve assay (HRM) etc have been developed to detect the efficiency of the induced mutations. However, these assays have some limitations in that they either require specific sequences in the target sites or are unable to generate sequencing-ready mutant DNA fragments or unable to distinguish induced mutations from natural nucleotide polymorphism. Here, we developed a simple PCR-based protocol for detecting indel mutations induced by TALEN and Cas9 in zebrafish. We designed 2 pairs of primers for each target locus, with one putative amplicon extending beyond the putative indel site and the other overlapping it. With these primers, we performed a qPCR assay to efficiently detect the frequencies of newly induced mutations, which was accompanied with a T-vector-based colony analysis to generate single-copy mutant fragment clones for subsequent DNA sequencing. Thus, our work has provided a very simple, efficient and fast assay for detecting induced mutations, which we anticipate will be widely used in the area of genome editing.

## Introduction

The past decade has witnessed remarkable advances in genome editing. A series of genome editing tools such as ZFNs, TALENs and Cas9 have been developed that enable investigators to manipulate virtually any gene in a wide range of cell types and organisms. Both ZFNs and TALENs have almost the same mode of action with their DNA recognition domains interacting with nucleotides and nuclease catalytic domains producing DNA double strand breaks (DSB). In the CRISPR/Cas9 system, a Cas9 protein is guided by a synthetic single guide RNA (sgRNA) consisting of a fusion of crRNA and tracrRNA to the target locus to produce cleavage at a PAM motif (NGG) [1]. The induced DSBs can be repaired by the NHEJ system, which in turn generates various mutations including insertion, deletion or even translocation at the target loci [2]. These technologies have been successfully applied in eukaryotic cells and model organisms for the disruption of target genes [3]. However, these genome editing tools are not perfect and about 40–60% of the target loci cannot be efficiently mutated, which calls for sensitive and reliable methods to evaluate the effectiveness of specific targets.

Currently, several assays have been developed to detect the mutations of target loci. The most frequently used assay is the hetero-duplex analysis, which utilizes SURVEYOR nuclease [4], Cel-I [5], [6] or T7E1 [7], [8], [9] to recognize and cut the hetero-duplex of mutant and wild-type DNA fragments containing mismatched base pairs. In addition, the hetero-duplex can be analyzed by high resolution melt curve analysis (HRMA) [10], [11], [12], [13]. Restriction enzyme assay is another frequently used method to evaluate TALEN and Cas9 activity when a target region contains a suitable restriction enzyme site [14], [15]. Furthermore, the LacZ recovery/disruption assay has been developed to assess the frame shift mutation resulted in the recovery of *lacZ* activity by blue/white colony screening [16]. However, current assays have their limitations in that they either require specific sequence in the target sites or are unable to generate sequencing-ready mutant DNA fragments or unable to distinguish induced mutation from natural nucleotide polymorphism.

To overcome these limitations, we have developed a simple PCR-based technique to detect the induced mutations. It is well known that the mutant positions induced by these nucleases are largely predictable, as ZFNs and TALENs induce genomic DSBs in the center of spacer region between their DNA recognition sites and Cas9 induce DSBs in proto-spacer adjacent motif (PAM) [1]. Taking advantage of this, we designed 2 pairs of primers for each TALEN or Cas9 target site, with one putative amplicon extending beyond the putative indel site and the other overlapping it. Thus, the newly introduced mutations will disrupt the amplification of the later amplicon but will not affect that of the first one. By using quantitative PCR (qPCR), we demonstrated that the efficiency of desired mutations could be quantitatively evaluated. And coupled with a T-vector-based mutant colonies identification assay (T-CIA), it could easily generate single-copy mutant DNA fragments for subsequent sequencing. Thus, our work provides a very simple, efficient and fast assay for detecting induced mutations, which we anticipate will be widely used in the area of genome editing.

## Materials and Methods

### Ethics Statement

The treatment and handling of zebrafish was performed under general directives on the protection of animals used for scientific purposes and following operating procedures approved by Sichuan Animal Care and Use Committee, Permit Number: SYXK (Chuan) 2008-119. Detailed treatment of adult zebrafish and embyos were described in the “DNA isolation and template preparation” section.

### Zebrafish Care

All the Zebrafish used in our experiment were AB strain. Adult fish and embryos were raised and maintained as described Westerfield et al [17], [18]. Embryos were cultured in 10 cm petri dishes at 28.5°C. Adult zebrafish were maintained in the standard tank of an automatic fish housing system (ESEN, China). Healthy 3–5 months old adult female and male fish were maintained in separate tanks and mated once a week.

### TALEN Target Site Design and Unit Assembly

TALEN target sites were designed with an online tool, TALE-NT (https://tale-nt.cac.cornell.edu/). Then, TALEN units were assembled using methods described in *Huang* et al. [19] and Zu et al. [20]. Briefly, the units were assembled to construct the TALE repeats according to the target sequences by several rounds of digestion/ligation cycles. Each round of digestion/ligation cycle, the recombinant vectors were transformed into *Escherichia coli* Top10 for amplification. The constructed repetitive TALE constructed fragments were then digested and inserted into TALEN expression vectors pXt7-TALEN upstream of *FokI*. The TALEN target sites were listed in Table S1 and Figure S1.

### Cas9 Target Site Design and Vector Construction

Cas9 target sites were designed with an online tool, ZIFIT Targeter (http://zifit.partners.org/zifit/Introduction.aspx). The vectors, pCS2-Cas9 and pT7-gRNA, for making Cas9 mRNA and chimera guide RNA were kind gifts from Jingwei Xiong and Bo Zhang (Peking University). The target site sequences were in vitro synthesized and inserted between the T7 promoter and guide RNA (sgRNA) scaffold sequence of pT7-gRNA [21]. The Cas9 target sites were listed in Table S1 and Figure S1.

### mRNA Synthesis

For mRNA synthesis, both the TALEN and Cas9 expression vectors were linearized and then transcribed in vitro with the mMESSAGE mMACHINE T7 ULTRA kit (Ambion) following manufacturer’s instructions. RNAs were purified by LiCl acetate precipitation and re-dissolved in RNase-free water. The RNA quality and concentration were analyzed by electrophoresis and nucleic acid spectrometer respectively.

### Microinjection of Zebrafish Embryos

One-cell stage embryos were collected for microinjection by using an electronically regulated air-pressure micro-injector (Harvard Apparatus, NY, PL1-90). For TALEN injection, equal amounts of the Left and Right arm mRNAs were mixed and injected together into the yolk of the embryos. For CRISPR/Cas9 injection, Cas9 mRNA and gRNA were mixed at a ratio of 5∶3.

### DNA Isolation and Template Preparation

Genomic DNA was extracted using the alkaline lysis method. For adult zebrafish DNA extraction, tails were cut from fish anaesthetized with 0.4 mg/ml tricaince solution (Sigma-Aldrich), and the fish were put back to the tank immediately after tail cutting. For embryonic DNA extraction, 5 embryos at 2 days post fertilization were pooled into each PCR tube. Embryos or tail fins were lysed in 30 µl alkaline lysis buffer (25 mM NaOH, 0.2 mM EDTA) and heated at 95°C for 10 minutes. Then, DNA solution was neutralized by adding 1/10 volume neutralization buffer (40 mM Tris-HCl, pH 8.0). Samples were spun at 10,000 rpm for 5 minutes and the supernatant was transferred into new tubes.

### qPCR Analysis for Identifying Mutations

We analyzed a panel of published mutation sites induced by TALEN and Cas9, and found that most mutations affected a 4 to 5 base region around the DSBs. Accordingly, we designed 2 pairs of primers: the first pair of primers were designed to prime outside the DSBs and the other flanked the DSBs at the 3′ most nucleotides. Thus, newly induced mutation will disrupt the amplification of the latter pair of primers. Before performing qPCR, we ran an ordinary PCR to obtain high quality DNA fragments using the outside primers, and these fragments were quantified and adjusted to equal amounts for subsequent qPCR. qPCR was performed with SsoAdvanced SYBR Green Supermix (Bio-Rad) by using a Bio-Rad CFX96 Real-Time system. All the primers were listed in Table S2. The qPCR conditions were programmed as follows: 40 cycles of 10 s at 95°C and 20 s at 60°C after initial denaturing for 30 s at 95°C.

### Isolation of Monocolonies Harboring Induced Mutations

The products from outside primers of qPCR were directly inserted into T-clone vector pMD19-T (Takara) and transformed into *Escherichia coli* Top10 for white-blue screening. Then the white colonies were analyzed by PCR using primers as described in Table S2. Those colonies that could not generate inside PCR fragments were the putative mutant ones which were further analyzed by DNA sequencing.

## Results and Discussion

Mutations caused by TALEN or Cas9 are largely predictable as the induced mutant loci were produced at a fixed target sequence. Most of the indel mutations occurred at the DSBs sites, referred as mutational hot spot regions (MHS). An analysis of several hundreds of the published mutant sequences revealed that most TALEN induced mutations affected the middle 4–5 base pairs of the spacer regions and Cas9 induced mutations affected the 4 base pairs upstream of the PAM (−4 to −1, counting 5′ to 3′ from the PAM). Specifically, in TALEN-induced mutations, 70.18% affected all the 4–5 base pairs, 11.47% affected 3 of them, 7.34% affected 2 of them, and 6.57% affected 1 of them (n = 654) (Document S1). In Cas9 induced mutations, 42.67% affected all the 4 base pairs, 17.48% affected 3 of them, 14.91% affected 2 of them, and 20.31% affected 1 of them (n = 389) (Document S2).

**Figure 1:**
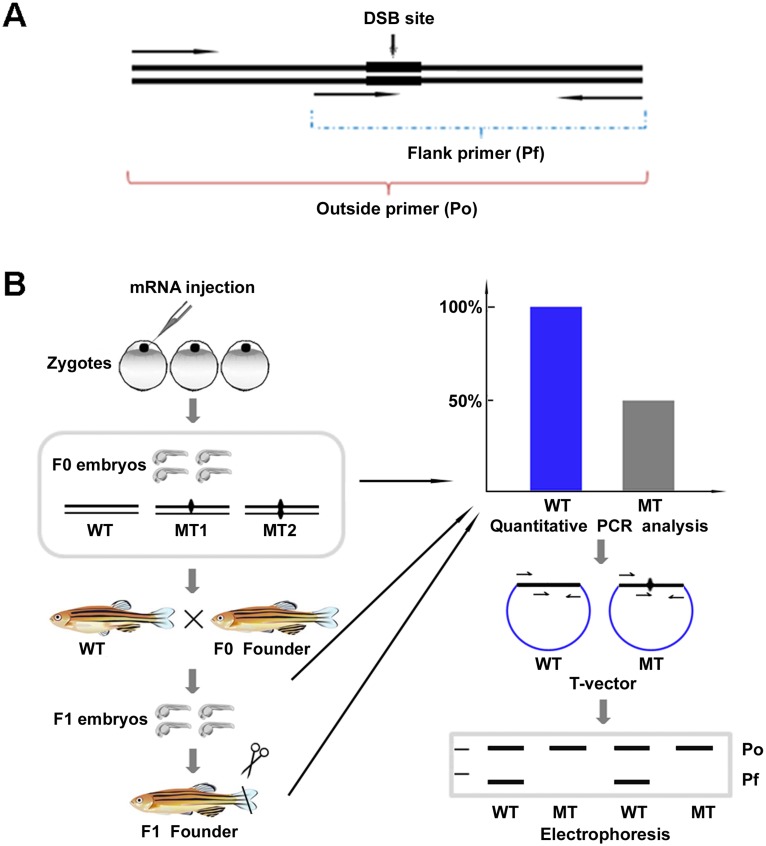
Schematic diagrams showing a PCR-based protocol for identifying mutations induced by TALEN and Cas9 in zebrafish. (A) Primers designed for detecting mutations in target site. (B) Procedure for identifying induced mutation in zebrafish. WT, wild-type. MT, mutant.

Because a highly complementary 3-prime end of the PCR primer is critical for amplification, mismatches in the 3′ end will greatly reduce the amplification efficiency. This phenomenon is widely used in PCR-based genotyping methods for detecting genetic polymorphism or point mutation [22], [23]. Theoretically, the induced indel mutations will cause complete or partial loss of the PCR amplification if the 3′ end of primers were designed to exactly cover MHSs. In view of this hypothesis, we designed a simple PCR based protocol to detect the expected mutations (Figure 1). As shown in Figure 1A and Table S2, one pair of primers were designed to prime outside the MHS, referred as Primer outside (Po), and the other flanked the MHS at the 3′ most nucleotides, referred as Primer flank (Pf). Thus, Po can amplify both wild-type and mutant alleles, while Pf can only amplify wild-type alleles, and any newly arising mutation will disrupt the amplification of the Pf. Technically, mutation frequency can be easily calculated by the decreased ratio between Pf amplicons and Po amplicons.

**Figure 2:**
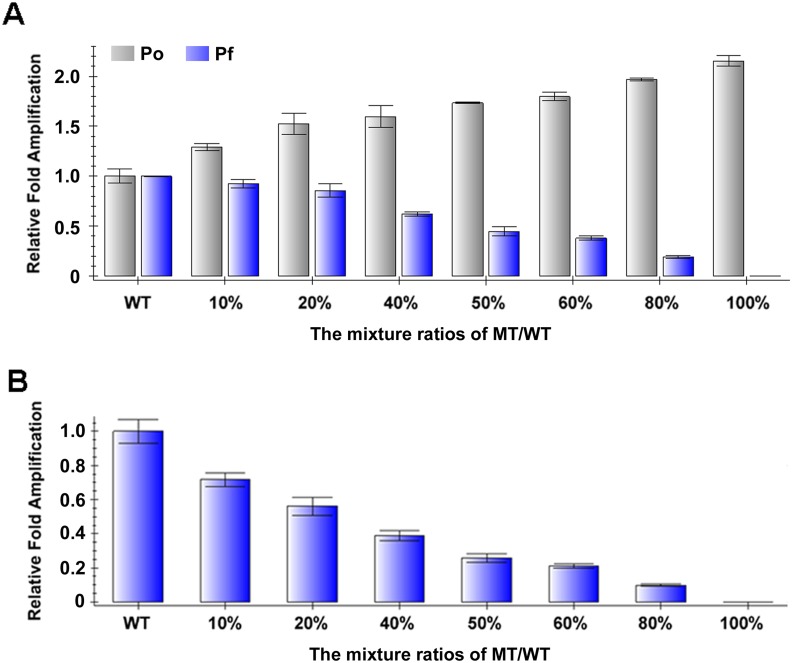
Determination of known mutations with qPCR. (A) Relative levels of Po or Pf PCR products. The amounts of Po or Pf PCR products of mixed templates were compared to the amount of corresponding PCR products of pure wild-type template. (B) Relative ratios between Po PCR products and Pf PCR products. The ratios between Po PCR products and Pf PCR products of mixed templates were compared to the ratio of wild-type template. WT, wild-type. MT, mutant.

As a first step to test the hypothesis, we examined whether this method could identify and quantify an already established 4 bp deletion mutation in the zebrafish *ldlr* gene. We diluted the mutant and wild-type templates to equal concentration and mixed them in various ratios ranging from 0% to 100%, herein referred as putative ratios. Then we used *ldlr*-Po primers that are mutation insensitive as an internal control and *ldlr*-Pf primers that are mutation sensitive as a test. As expected, the amplification efficiencies of *ldlr*-Pf were gradually reduced along with the increasing ratios of mutant templates in the mixtures (Figure 2A). More importantly, these data showed that qPCR could detect at least 10% mutation in the template which is much lower than the efficiency of most reported TALEN and Cas9 induced mutations. However, the amplification efficiencies of Po primers were gradually increased along with the increasing ratios of mutant templates in the mixtures (Figure 2A), and the calculated ratios of mutant fragments by qPCR were greater than the putative ratios (Figure 2B). The deviation could be caused by inaccurate measurement of the concentration of original templates or from errors during dilution and mixing. Nevertheless, this is not an issue when using this method in detecting induced mutations, because we do not need to mix the wild-type and the putative mutant templates in such situations.

**Figure 3:**
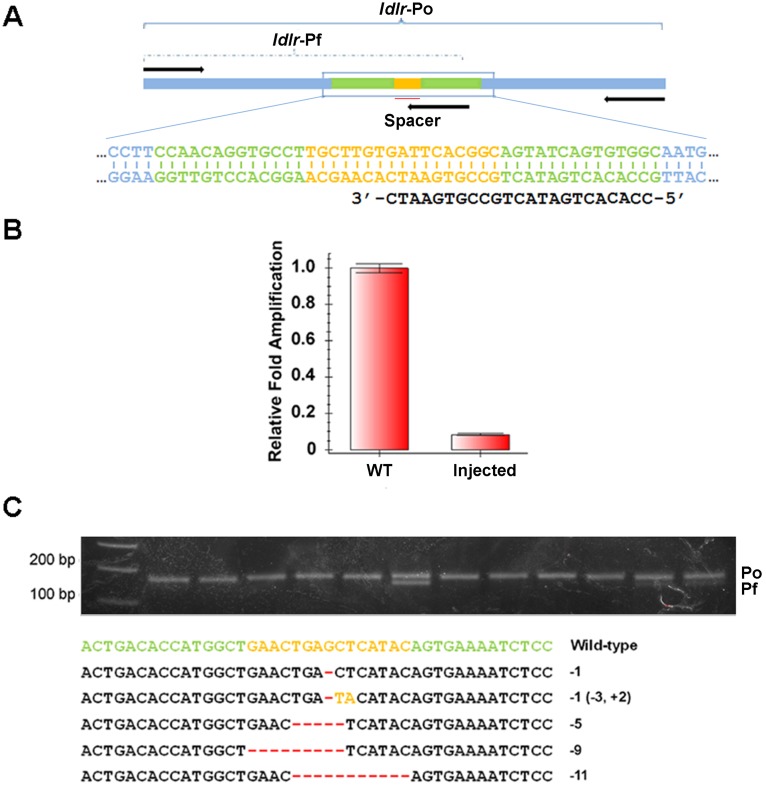
Identification of mutations induced by *ldlr* TALEN. (A) A schematic diagram showing primers for detecting mutations in TALEN target sites of *ldlr* gene. (B) The relative amplification efficiency of *ldlr* Pf primers to Po primers. *ldlr* TALEN mRNAs injection dramatically reduced the amplification efficiency of *ldlr* Pf primers. 5 embryos from the wild-type group or the *ldlr*-TALEN-injected group were pooled and analyzed by qPCR, and 3 pools of each group were analyzed in an independent experiment. Similar results were obtained in three independent experiments. (C) Identification of a mutation in a single allele with T-CIA. Upper panel was an agarose electrophoresis result of PCR products amplified with *ldlr* Po and Pf primers. Lower panel listed the sequences of mutant colonies identified in upper panel.

**Figure 4:**
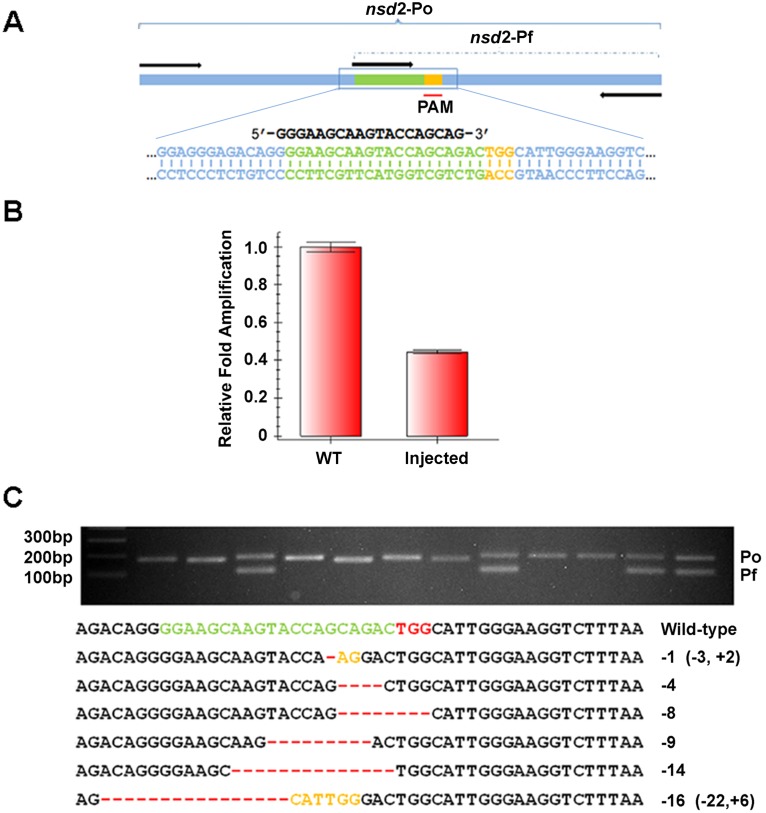
Identification of mutations induced by *nsd2* Cas9. (A) A schematic diagram showing primers for detecting mutations in Cas9 target site of *nsd2* gene. (B) The relative amplification efficiency of *nsd2* Pf primers to Po primers. *nsd2* Cas9 mRNA injection dramatically reduced the amplification efficiency of *nsd2* Pf primers. 5 embryos from the wild-type or the *nsd2*-Cas9-injected group were pooled and analyzed by qPCR, and 3 pools of each group were analyzed in an independent experiment. Similar results were obtained in three independent experiments. (C) Identification of a mutation in a single allele with T-CIA. Upper panel was an agarose electrophoresis result of PCR products amplified with *nsd2* Po and Pf primers. Lower panel listed the sequences of mutant colonies identified in upper panel.

Next, we tested this method in detecting new mutations at several designed TALEN or Cas9 target sites. We injected one cell-stage zebrafish embryos with either TALEN mRNAs or an RNA mixture of Cas9 mRNA and sgRNAs. Genomic DNA was prepared from a pool of 5 injected or wild-type embryos and analyzed by qPCR. As shown in figure 3B, *ldlr* TALEN mRNA injection greatly reduced the amplification of the Pf primers but not that of the Po primers. The normalized efficiency of *ldlr* TALENs was about 91.6%. Figure 4B showed the qPCR results of a Cas9 targeting the *nsd2* gene, in which the normalized efficiency was about 44.1%. We also used this method to successfully test 2 additional TALEN or Cas9 target sites, *apoeb* and *nsd3* (Figure S2B and S1B). At the same time, we also confirmed the presence of indel mutations in *nsd2* and *nsd3* loci with restriction endonuclease assays, and observed similar mutant efficiency (Figure S3). Thus, these results established that the qPCR assay can reliably calculate the efficiency of TALEN or Cas9 induced mutations.

In order to further confirm the reliability of this method and to generate single-copy mutant DNA fragments for subsequent sequencing, we cloned the PCR products of the injected embryos into T-cloning vectors, pMD19-T. About 10 colonies of each target site were examined by ordinary PCR using the corresponding Po and Pf primers. As shown in Figure 3C, 4C and Figure S1C, all the white colonies can be amplified by the Po primers while some of them cannot be amplified by the Pf primers, indicating that these colonies harbored mutant DNA fragments. To confirm this, we selected several colonies that possess only Po fragment for sequencing and found that almost all the positive colonies had indel mutations in the target sites (Figure 3C and 4C). Together, our data suggested that this PCR-based assay is indeed affective and sensitive for both determining mutation efficiency and identifying mutant sequences.

Then, we tested if this method could be utilized to detect germ line transmitted mutations. TALEN or Cas9 mRNA injected embryos were raised to adulthood (referred as F0 fish) and outcrossed with wild-type fish. Pools of 5 F1 embyos were lysed and subjected to qPCR analysis. In Figure S4 A and C, we identified one out of four fish that had germ line mutation in the *nsd2* gene, and all 3 F0 fish examined had germ line mutation in *ldlr* gene. Again, the offspring of these F1 fish were raised to adulthood and were genotyped by qPCR using DNA extracted from tail fins (Fig. S4 B and D). Interestingly, all the F1 fish analyzed were heterozygous mutant for *nsd2* or *ldlr* gene. Then the mutant allele of each F1 fish was analyzed by T-CIA (data not shown). Thus, this method is practical for detecting germ line transmitted mutations as well.

In summary, we have developed a simple PCR-based method for detecting indel mutations induced by TALEN and Cas9 in zebrafish. We demonstrated that qPCR assay could specifically detect indel mutations arising at designed target sites, and results from this assay should not be disturbed by natural polymorphism in the genome. In addition, this assay does not require a restriction site, thus it does not bias the choice of a target site. Furthermore, we also provided a T-vector-based assay for identifying mutant sequences of the target loci. However, theoretically, this assay is less efficient in detecting mutations in regions containing repeated nucleotides, especially mono-nucleotide repeats. It is also not efficient in detecting mutations that do not happen in the predicted MHS. But besides these situations, this assay is highly efficient. Considering that it could be used in the entire procedure of nuclease-mediated genome editing, including the evaluation of the efficiency of a given TALEN or Cas9, and the identification of founder or F1 fishes, we hope this assay will be widely used in the field of in genome editing technologies.

## Supporting Information

Figure S1 **The designs of TALENs and Cas9 targeting regions in zebrafish.** (A) and (B) were using TALENs to disrupt *ldlr* and *apoeb* genes respectively. (C) and (D) were using Cas9 to disrupt *nsd2* and *nsd3* genes respectively.
(TIF)

Figure S2 **qPCR identify mutations induced by NSD3 Cas9.** (A) A chematic diagram showing primers for detecting mutations in Cas9 target site of *nsd3* gene. (B) The relative amplification efficiency of *nsd3* Pf primers to Po primers. *nsd3 cas9* mRNAs injection dramatically reduced the amplification efficiency of *nsd3* Pf primers. (C) Identification of mutation in single allele with T-CIA. Upper panel was agarose electrophoresis result of PCR products amplified with *nsd3* Po and Pf primers. Lower panel listed the sequences of mutant colonies identified in upper panel. Every 5 embryos served as a pool for qPCR. The Data in B were obtained from 3 independent experiments, with 3 replicates for each pool.
(TIF)

Figure S3 **qPCR identify mutations induced by TALEN.** (A) A chematic diagram showing primers for detecting mutations in TALEN target site of *apoeb* gene. (B) The relative amplification efficiency of *apoeb* Pf primers to Po primers. *apoeb* TALEN mRNAs injection dramatically reduced the amplification efficiency of *nsd3* Pf primers. (C) A list of the mutant sequences of TALEN target site in *apoeb* gene. Every 5 embryos served as a pool for qPCR. The Data in B were obtained from 3 independent experiments, with 3 replicates for each pool.
(TIF)

Figure S4 **The calculated efficiencies determined by qPCR were comparable to that by restriction endonuclease assays.** (A) Cas9 induced mutations of *nsd2* and *nsd3* were detected by qPCR. As a comparsion, *nsd2* (B) and *nsd3* (C) were identified by restriction endonuclease assays as well. The results showed that these two methods almost have the same mutant efficiencies. Every 5 embryos served as a pool for qPCR. The Data were obtained from 3 independent experiments, with 3 replicates for each pool.
(TIF)

Figure S5 **Using qPCR to detect germ line transmitted mutations.** (A) Pools of every five F1 embryos from *nsd2* F0 zebrafish outcrossed with wild type zebrafish were identified by qPCR. Result indicated that zebrafish 2# had germ line transmitted mutation. (B) Genomic DNAs from tail fins of adult descendants of line 2# were detected by qPCR. Result showed that all the F1 zebrafish are mutant. (C) Genomes *of* F1 embryos from *ldlr* F0 zebrafish outcrossed with wild type zebrafish were checked by qPCR. All F0 zebrafishes had germ line transmitted mutation. (D) Genome DNAs from tail fins of adult F1 zebrafish derived from zebrafish labeled 1 were detected by qPCR. All the F1 zebrafish are mutant. Ever 5 embryos or each tail fin served as a pool for qPCR. The Data were obtained from 3 independent experiments, with 3 replicates for each pool.
(TIF)

Table S1 **The target sites of TALENs and Cas9.**
(DOCX)

Table S2 **Primers for identifying mutations induced by TALENs and Cas9.**
(DOCX)

Document S1 **Sequence analysis of TALENs induced mutations.**
(DOCX)

Document S2 **Sequence analysis of Cas9 induced mutations.**
(DOCX)
