# Supplementary figures and images for: A PCR Based Protocol for Detecting Indel Mutations Induced by TALENs and CRISPR/Cas9 in Zebrafish
Source: PLoS One. 2014 Jun 5;9(6):e98282. doi: 10.1371/journal.pone.0098282 (PMC4046980; doi:10.1371/journal.pone.0098282)

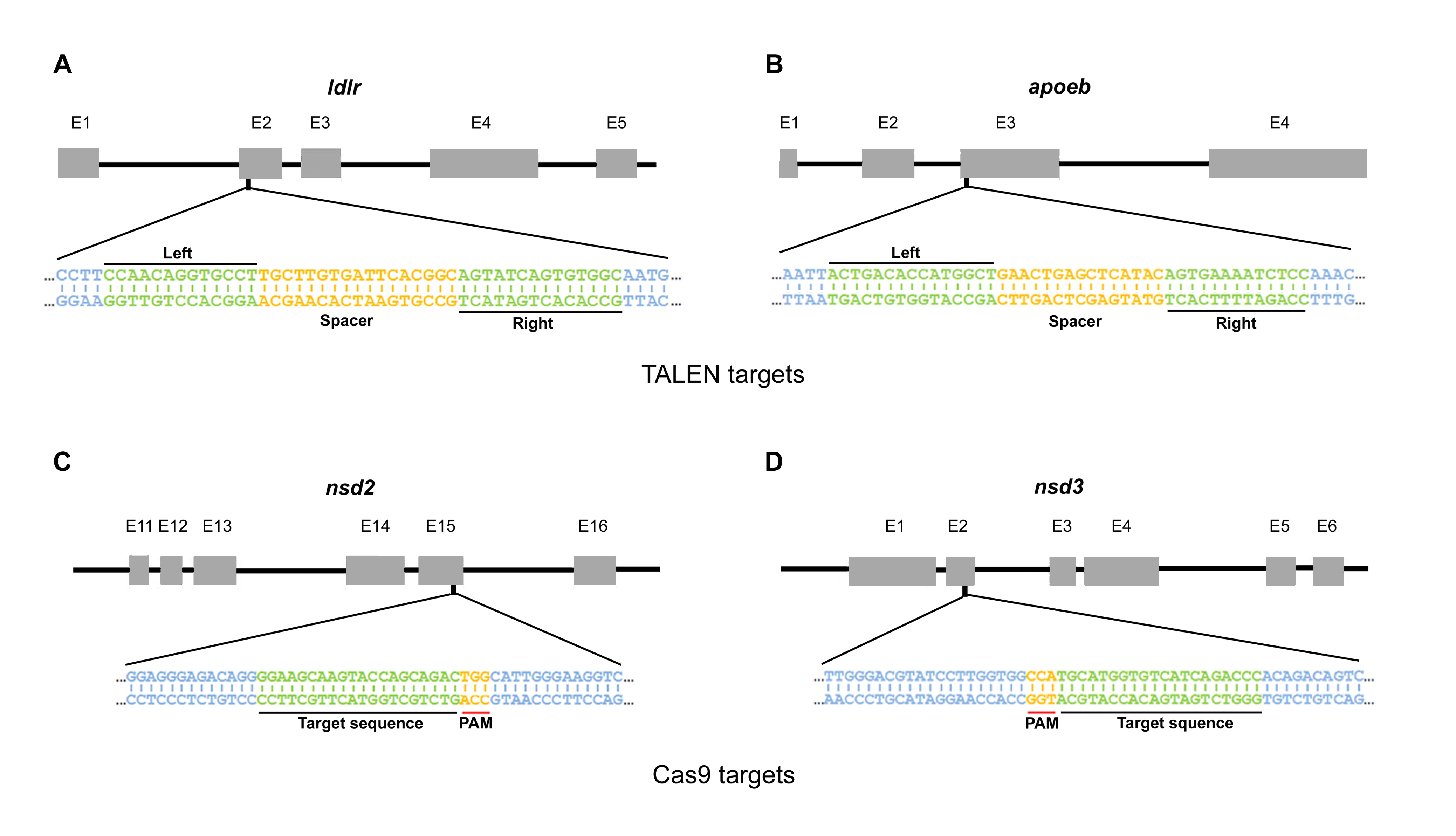

Supplement: Figure S1 — The designs of TALENs and Cas9 targeting regions in zebrafish. (A) and (B) were using TALENs to disrupt ldlr and apoeb genes respectively. (C) and (D) were using Cas9 to disrupt nsd2 and nsd3 genes respectively. (TIF) [file pone.0098282.s001.tif]

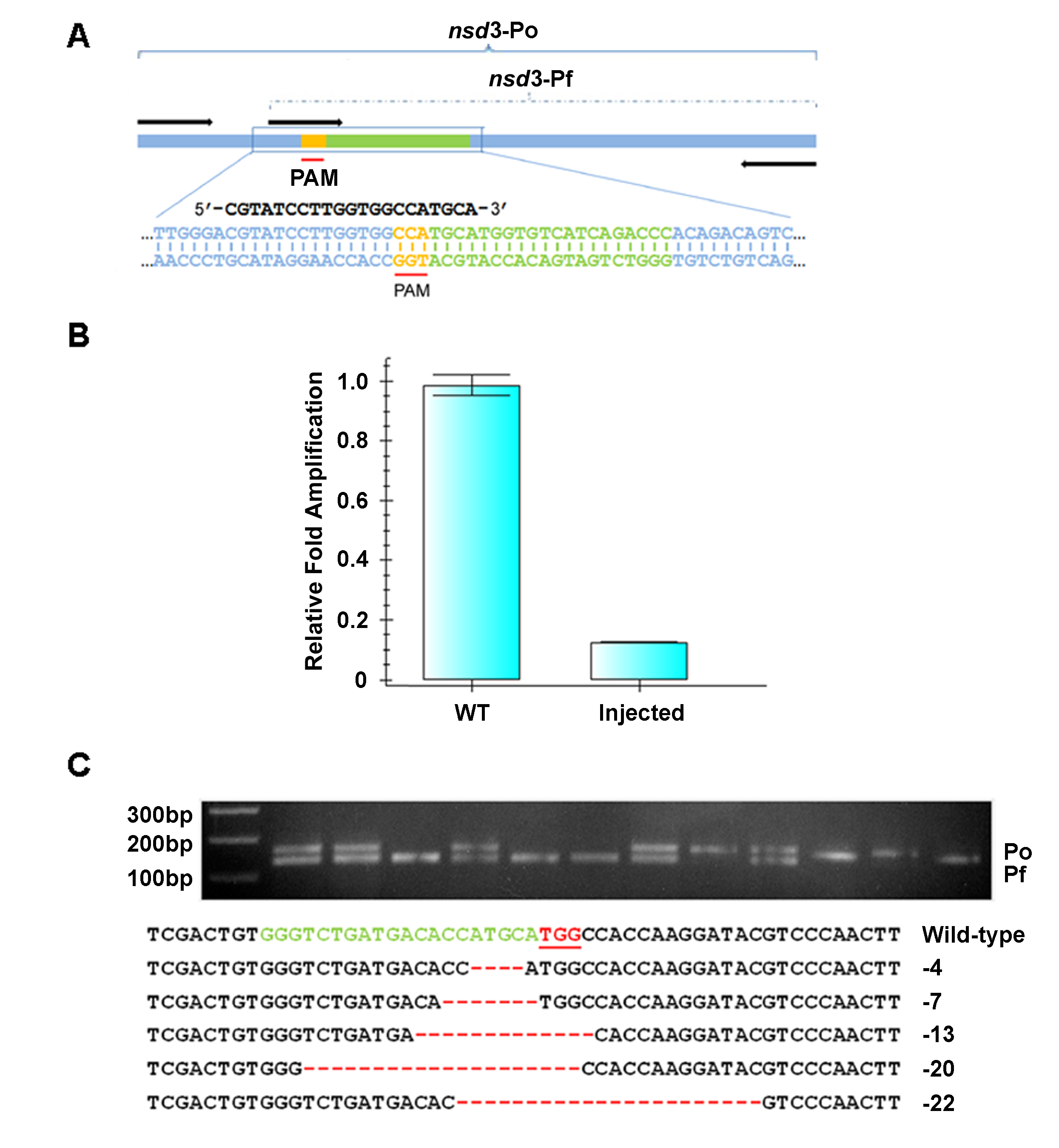

Supplement: Figure S2 — qPCR identify mutations induced by NSD3 Cas9. (A) A chematic diagram showing primers for detecting mutations in Cas9 target site of nsd3 gene. (B) The relative amplification efficiency of nsd3 Pf primers to Po primers. nsd3 cas9 mRNAs injection dramatically reduced the amplification efficiency of nsd3 Pf primers. (C) Identification of mutation in single allele with T-CIA. Upper panel was agarose electrophoresis result of PCR products amplified with nsd3 Po and Pf primers. Lower panel listed the sequences of mutant colonies identified in upper panel. Every 5 embryos served as a pool for qPCR. The Data in B were obtained from 3 independent experiments, with 3 replicates for each pool. (TIF) [file pone.0098282.s002.tif]

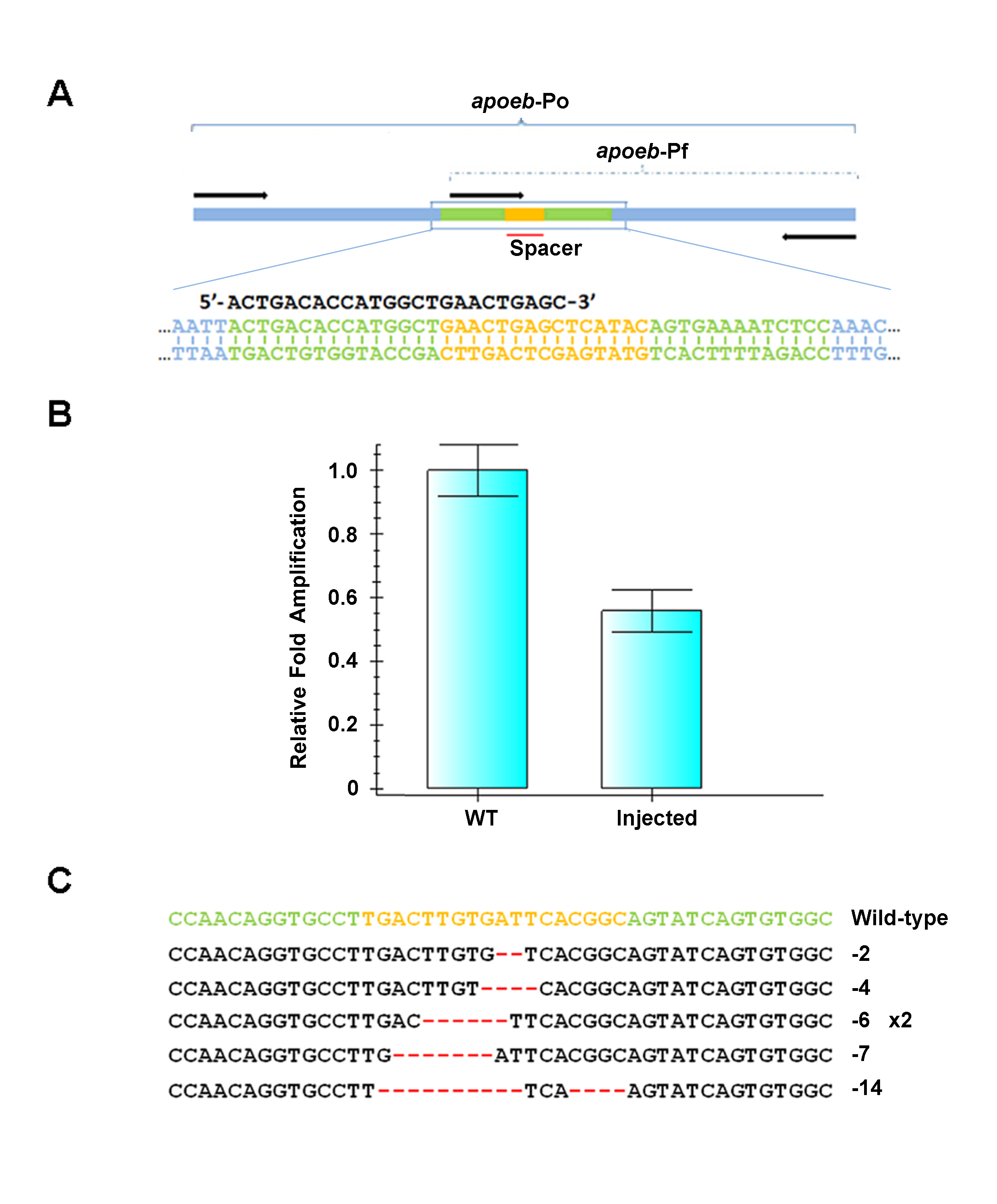

Supplement: Figure S3 — qPCR identify mutations induced by TALEN. (A) A chematic diagram showing primers for detecting mutations in TALEN target site of apoeb gene. (B) The relative amplification efficiency of apoeb Pf primers to Po primers. apoeb TALEN mRNAs injection dramatically reduced the amplification efficiency of nsd3 Pf primers. (C) A list of the mutant sequences of TALEN target site in apoeb gene. Every 5 embryos served as a pool for qPCR. The Data in B were obtained from 3 independent experiments, with 3 replicates for each pool. (TIF) [file pone.0098282.s003.tif]

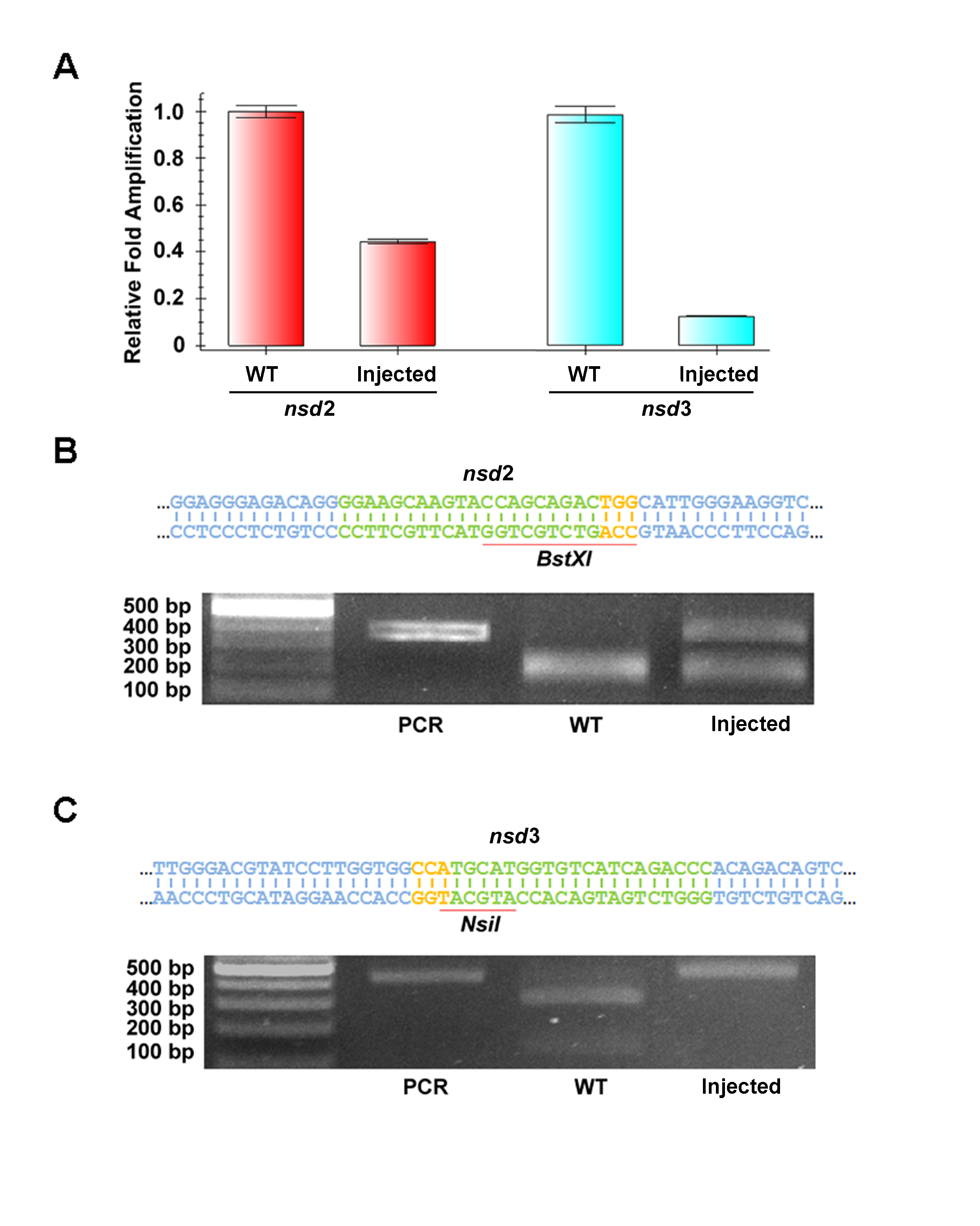

Supplement: Figure S4 — The calculated efficiencies determined by qPCR were comparable to that by restriction endonuclease assays. (A) Cas9 induced mutations of nsd2 and nsd3 were detected by qPCR. As a comparsion, nsd2 (B) and nsd3 (C) were identified by restriction endonuclease assays as well. The results showed that these two methods almost have the same mutant efficiencies. Every 5 embryos served as a pool for qPCR. The Data were obtained from 3 independent experiments, with 3 replicates for each pool. (TIF) [file pone.0098282.s004.tif]

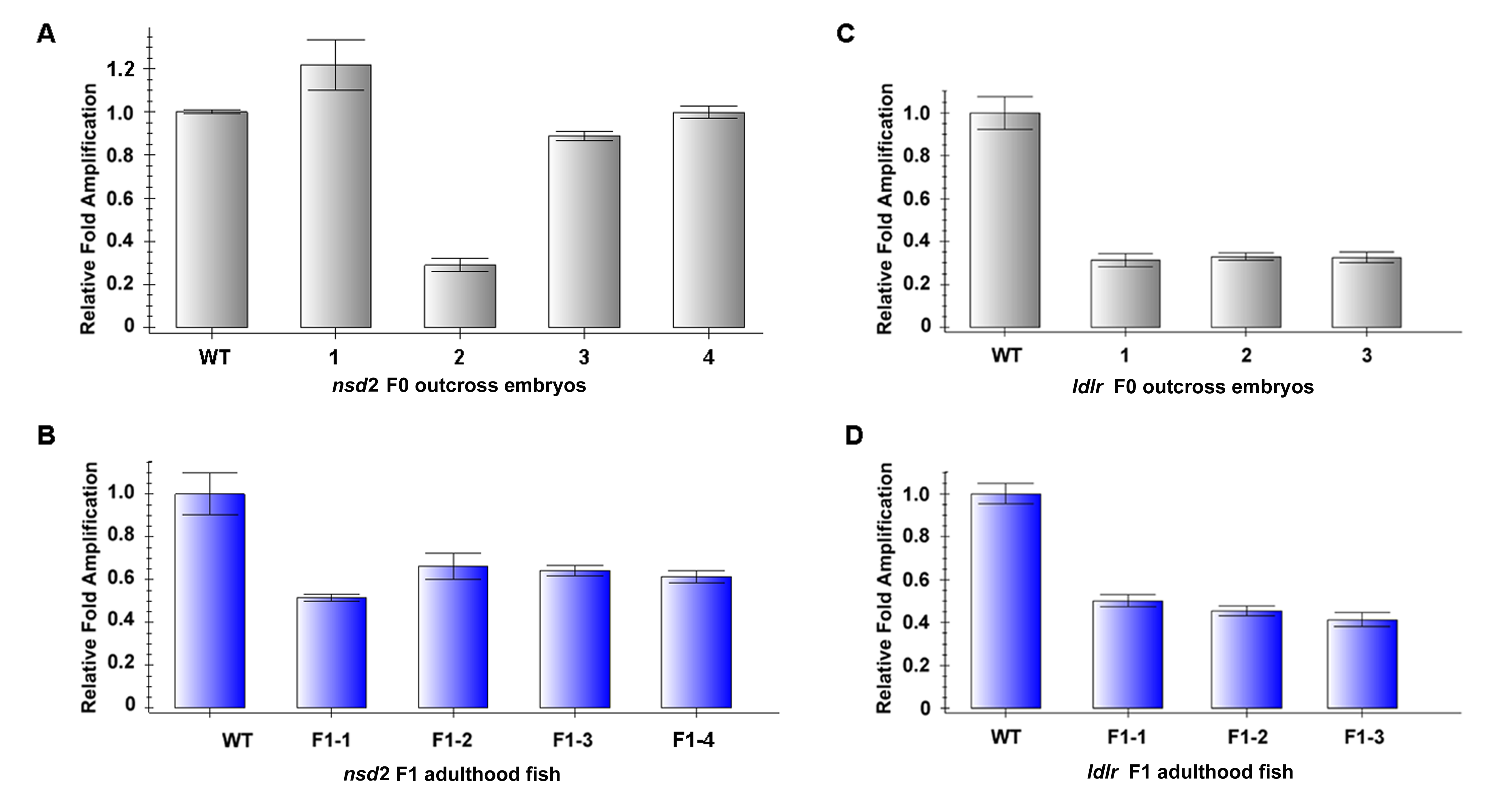

Supplement: Figure S5 — Using qPCR to detect germ line transmitted mutations. (A) Pools of every five F1 embryos from nsd2 F0 zebrafish outcrossed with wild type zebrafish were identified by qPCR. Result indicated that zebrafish 2# had germ line transmitted mutation. (B) Genomic DNAs from tail fins of adult descendants of line 2# were detected by qPCR. Result showed that all the F1 zebrafish are mutant. (C) Genomes of F1 embryos from ldlr F0 zebrafish outcrossed with wild type zebrafish were checked by qPCR. All F0 zebrafishes had germ line transmitted mutation. (D) Genome DNAs from tail fins of adult F1 zebrafish derived from zebrafish labeled 1 were detected by qPCR. All the F1 zebrafish are mutant. Ever 5 embryos or each tail fin served as a pool for qPCR. The Data were obtained from 3 independent experiments, with 3 replicates for each pool. (TIF) [file pone.0098282.s005.tif]
