# Supplementary material for: A PCR Based Protocol for Detecting Indel Mutations Induced by TALENs and CRISPR/Cas9 in Zebrafish
Source: PLoS One. 2014 Jun 5;9(6):e98282. doi: 10.1371/journal.pone.0098282 (PMC4046980; doi:10.1371/journal.pone.0098282)
Supplement: Table S1 — The target sites of TALENs and Cas9. (DOCX) [file pone.0098282.s006.docx]

Table S1 The target sites of TALENs and Cas9

| TALEN | |
| --- | --- |
| Gene | TALEN target sites |
| *apoeb* | Leftarm: TACTGACACCATGGCT  Rightarm: TGGAGATTTTCACT |
| *ldlr* | Leftarm: TCCAACAGGTGCCTT  Rightarm: TGCCACACTGATACT |
| CRIPR/CaS9 | |
| Gene | Primers for gRNA templates (the target sites are underlined ) |
| *nsd2* | *nsd2/*gRNA-F: TAATACGACTCACTATAGGAAGCAAGTACCAGCAGACGTTTTAGAGCTAGAAATAGC  *nsd2/*gRNA-R: AAAAGCACCGACTCGGTGC |
| *nsd3* | *nsd3/*gRNA-F: TAATACGACTCACTATAGGGTCTGATGACACCATGCAGTTTTAGAGCTAGAAATAGC  *nsd3/*gRNA-R: AAAAGCACCGACTCGGTGC |
