# Supplementary material for: A PCR Based Protocol for Detecting Indel Mutations Induced by TALENs and CRISPR/Cas9 in Zebrafish
Source: PLoS One. 2014 Jun 5;9(6):e98282. doi: 10.1371/journal.pone.0098282 (PMC4046980; doi:10.1371/journal.pone.0098282)
Supplement: Table S2 — Primers for identifying mutations induced by TALENs and Cas9. (DOCX) [file pone.0098282.s007.docx]

Table S2 Primers for identifying mutations induced by TALENs and Cas9

| Gene | Flank Primers (Pf) | Outside primers (Po) |
| --- | --- | --- |
| *ldlr* | *ldlr*-F: TCACAACGGACGAGGCGTGT  *ldlr*-R1: CGCCGTGATGCACTTTCCATTGCC | *ldlr*-F: TCACAACGGACGAGGCGTGT  *ldlr*-R2: CCACACTGATACTGCCGTGAATC |
| *apoeb* | *apoeb*-F1: 5’-ACTGTGACATCATTCCTGACCTGC-3’  *apoeb*-R: TGCTGCGTTCCTTAGCGTCGG | *apoeb*-F2: 5’-ACTGACACCATGGCTGAACTGAGC-3’  *apoeb*-R: TGCTGCGTTCCTTAGCGTCGG |
| *nsd2* | *nsd2*-F1: 5’-CCGTCTTCTTCTTTGGCTCT-3’  *nsd2*-R: 5’-CGGCTCTAATAATGCTGATATACA-3’ | *nsd2*-F2: 5’-AGGGAAGCAAGTACCAGCAG-3’  *nsd2*-R: 5’-CGGCTCTAATAATGCTGATATACA-3’ |
| *nsd3* | *nsd3*-F1: 5’-TGGTGACTTGGTGTGGGCA-3’  *nsd3*-R: 5’-GATGCAATGTGAATGGCCCC-3’ | *nsd3*-F2: 5’-CGTATCCTTGGTGGCCATGC-3’  *nsd3*-R: 5’-GATGCAATGTGAATGGCCCC-3’ |
