# Supplementary material for: A PCR Based Protocol for Detecting Indel Mutations Induced by TALENs and CRISPR/Cas9 in Zebrafish
Source: PLoS One. 2014 Jun 5;9(6):e98282. doi: 10.1371/journal.pone.0098282 (PMC4046980; doi:10.1371/journal.pone.0098282)
Supplement: Document S1 — Sequence analysis of TALENs induced mutations. (DOCX) [file pone.0098282.s008.docx]

**Sequence analysis of TALENs induced mutations**

目录

[**Reference 1**. 3](#_Toc385580792)

[**Reference 2** 5](#_Toc385580793)

[**Reference 3** 7](#_Toc385580794)

[**Reference 4** 10](#_Toc385580795)

[**Reference 5** 11](#_Toc385580796)

[**Reference 6** 13](#_Toc385580797)

[**Reference 7** 14](#_Toc385580798)

[**Reference 8** 15](#_Toc385580799)

[**Reference 9** 16](#_Toc385580800)

[**Reference 10** 17](#_Toc385580801)

[**Reference 11** 18](#_Toc385580802)

[**Reference 12** 18](#_Toc385580803)

[**Table 1** 21](#_Toc385580804)

**Reference 1:** Moore FE, Reyon D, Sander J, *et al*. (2012) DImproved Somatic Mutagenesis in Zebrafish Using Transcription Activator-Like Effector Nucleases (TALENs). PLoS ONE, 7(5): e37877.


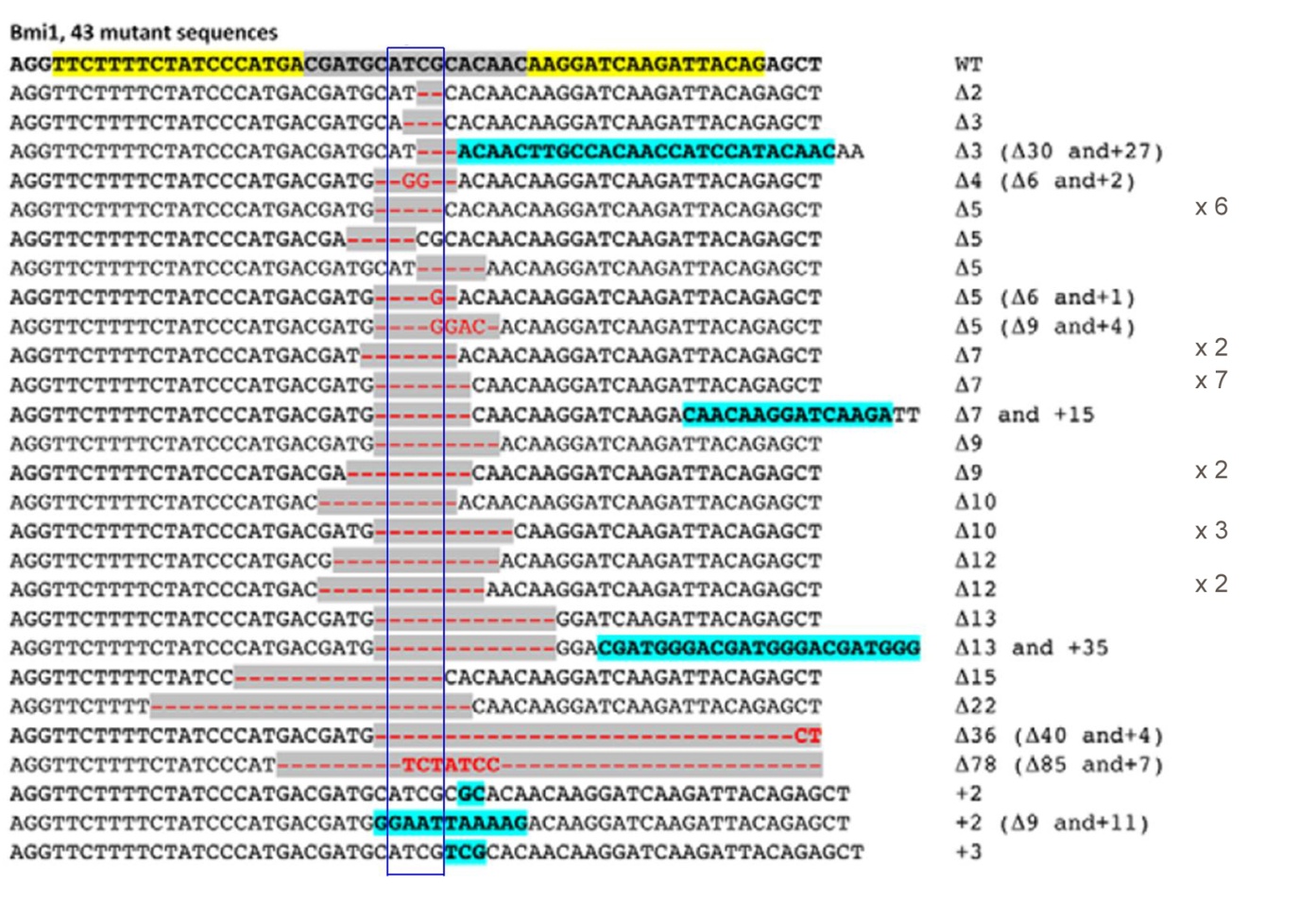
Mutation sequences: 43

Mutations including 4 or 5 bases: 36

Mutations including 3 bases: 1

Mutations including 2 bases: 4

Mutations including 1 bases: 0


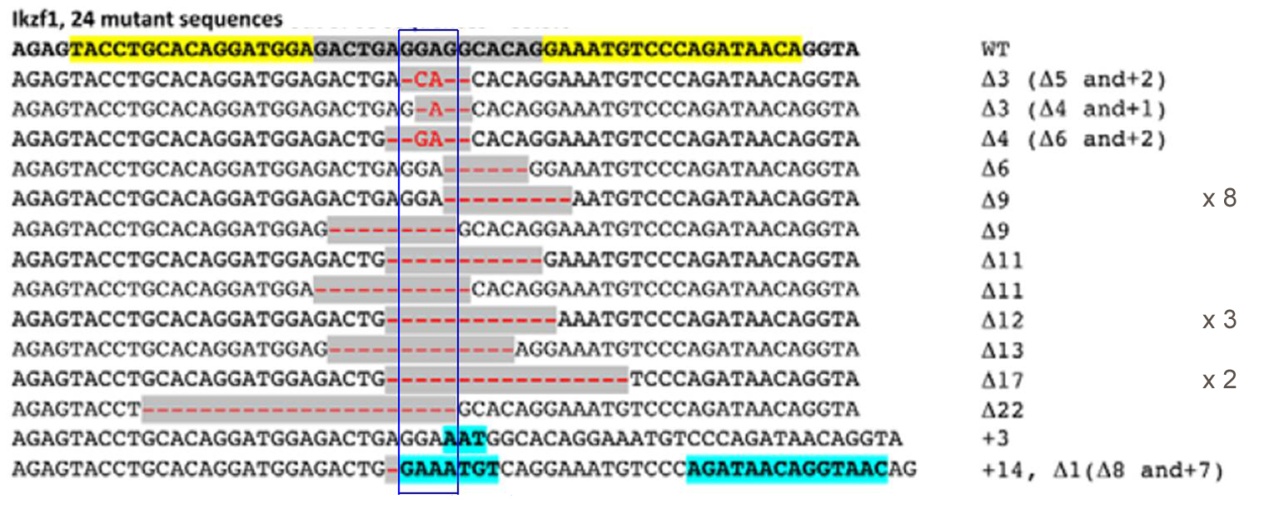
Mutation sequences: 24

Mutations including 4 or 5 bases: 13

Mutations including 3 bases: 1

Mutations including 2 bases: 0

Mutations including 1 base: 10


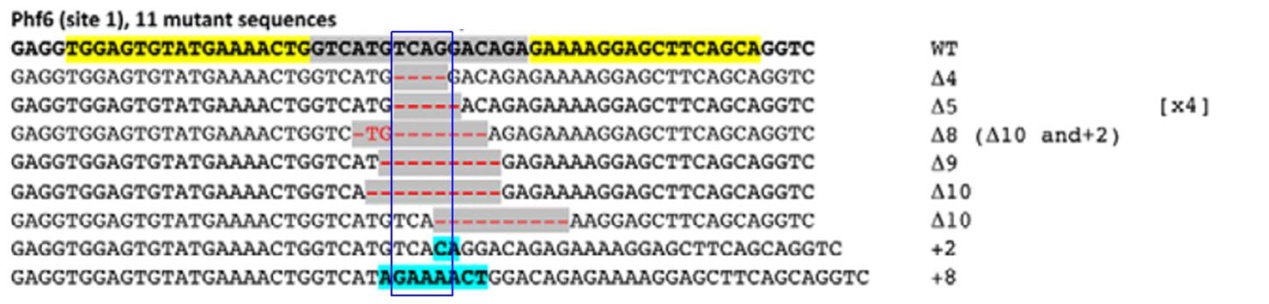


Mutation sequences: 11

Mutations including 4 or 5 bases: 9

Mutations including 3 bases: 0

Mutations including 2 bases: 0

Mutations including 1 base: 2


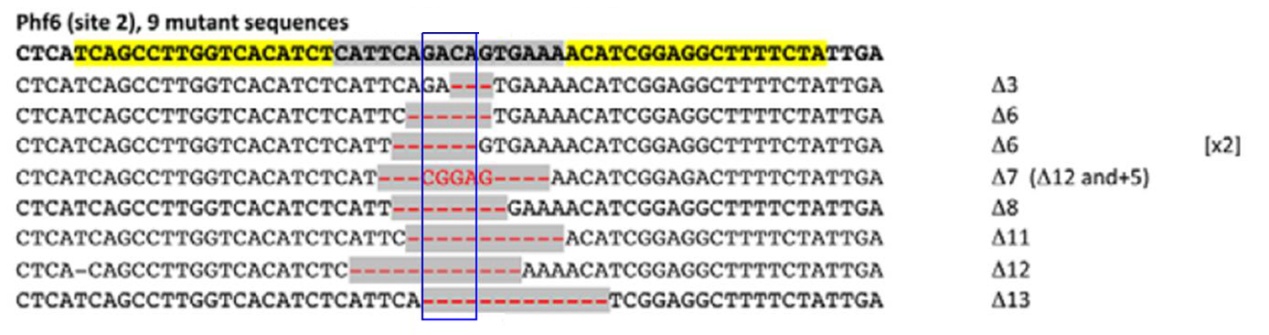


Mutation sequences: 9

Mutations including 4 or 5 bases: 8

Mutations including 3 bases: 0

Mutations including 2 bases: 1

Mutations including 1 base: 0


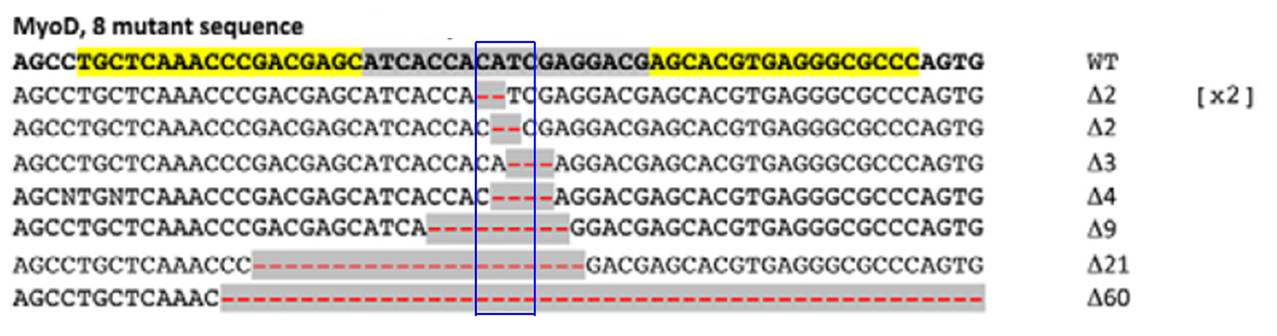


Mutation sequences: 8

Mutations including 4 or 5 bases:3

Mutations including 3 bases: 2

Mutations including 2 bases: 3

Mutations including 1 base: 0


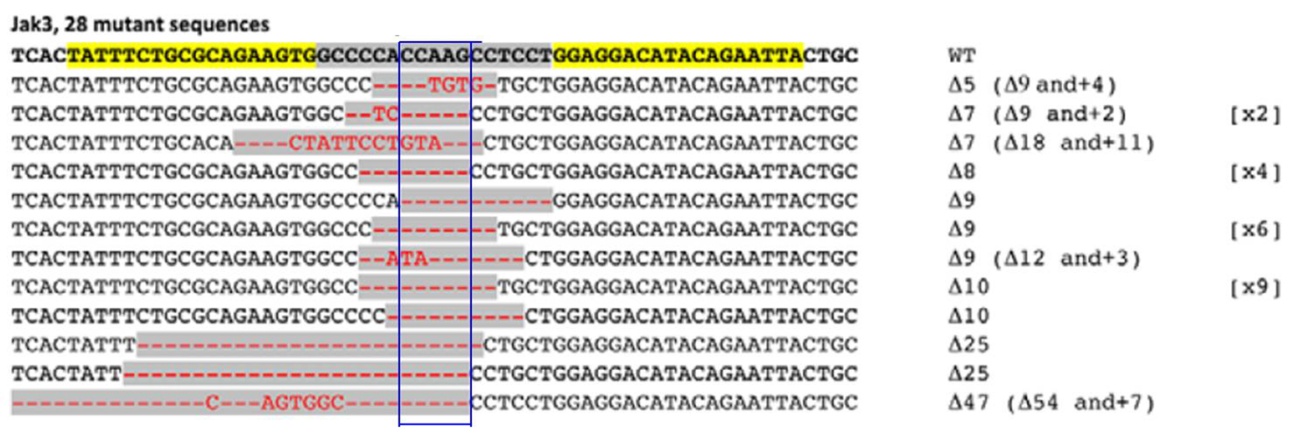


Mutation sequences: 29

Mutations including 4 or 5 bases: 29

Mutations including 3 bases: 0

Mutations including 2 bases: 0

Mutations including 1 base: 0

**Reference 2:** Dahlem TJ, Hoshijima K, Jurynec MJ, *et al*. (2012) Simple Methods for Generating and Detecting Locus-Specific Mutations Induced with TALENs in the Zebrafish Genome. PLOS Genetics, 8(8):e1002861


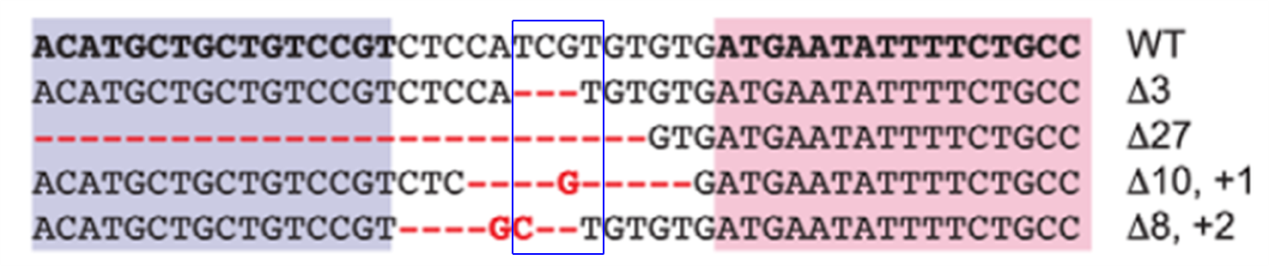


Mutation sequences: 4

Mutations including 4 or 5 bases: 2

Mutations including 3 bases: 2

Mutations including 2 bases: 0

Mutations including 1 base: 0


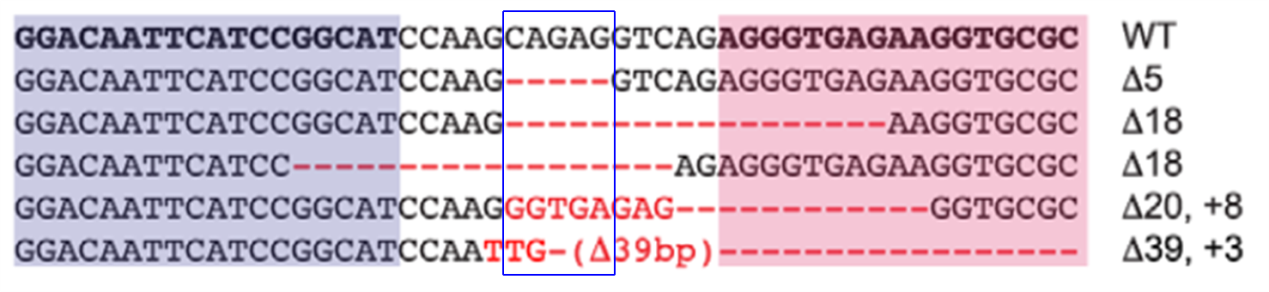


Mutation sequences: 5

Mutations including 4 or 5 bases: 5

Mutations including 3 bases: 0

Mutations including 2 bases: 0

Mutations including 1 base: 0


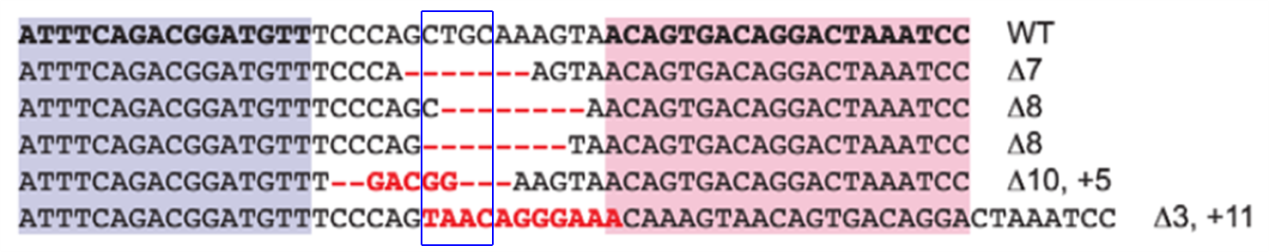


Mutation sequences: 5

Mutations including 4 or 5 bases: 4

Mutations including 3 bases: 1

Mutations including 2 bases: 0

Mutations including 1 base: 0


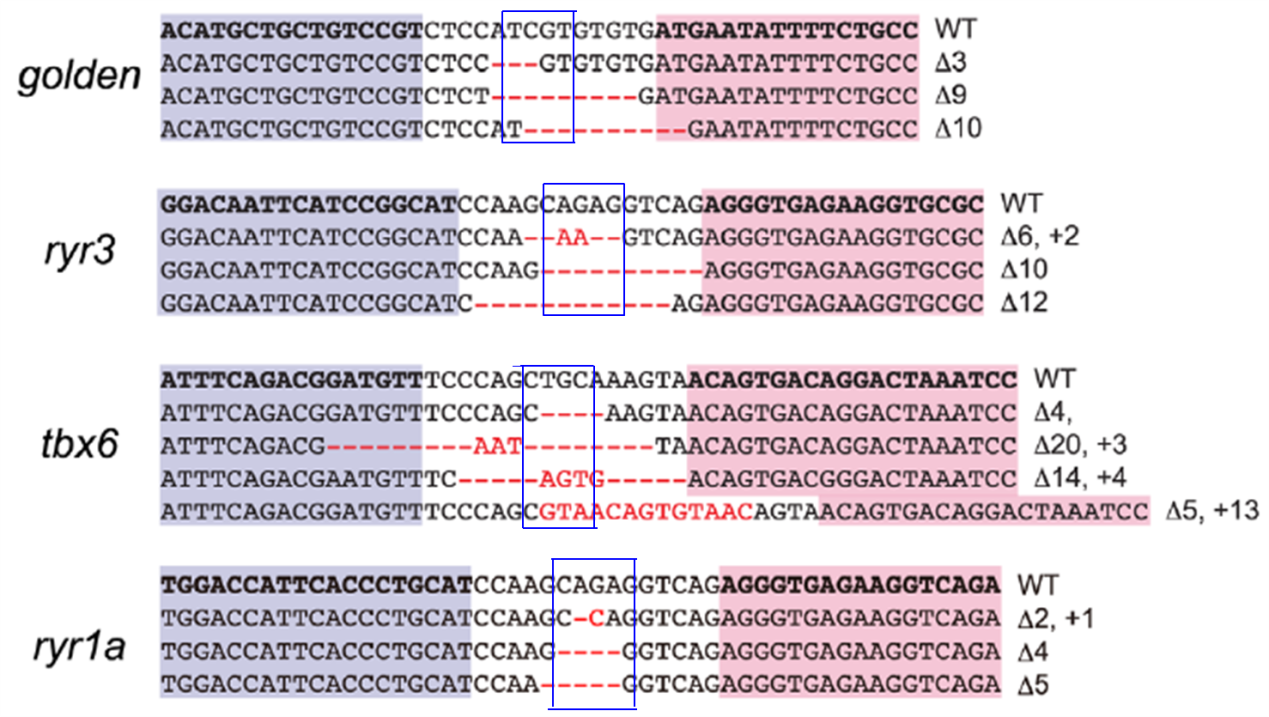


Mutation sequences: 13

Mutations including 4 or 5 bases: 8

Mutations including 3 bases: 3

Mutations including 2 bases: 2

Mutations including 1 base: 0

**Reference 3:** Reyon D, Tsai SQ, Khayter C, *et al*. (2012) FLASH Assembly of TALENs Enables High-Throughput Genome Editing. Nature biotechnology, 30(5): 460–465


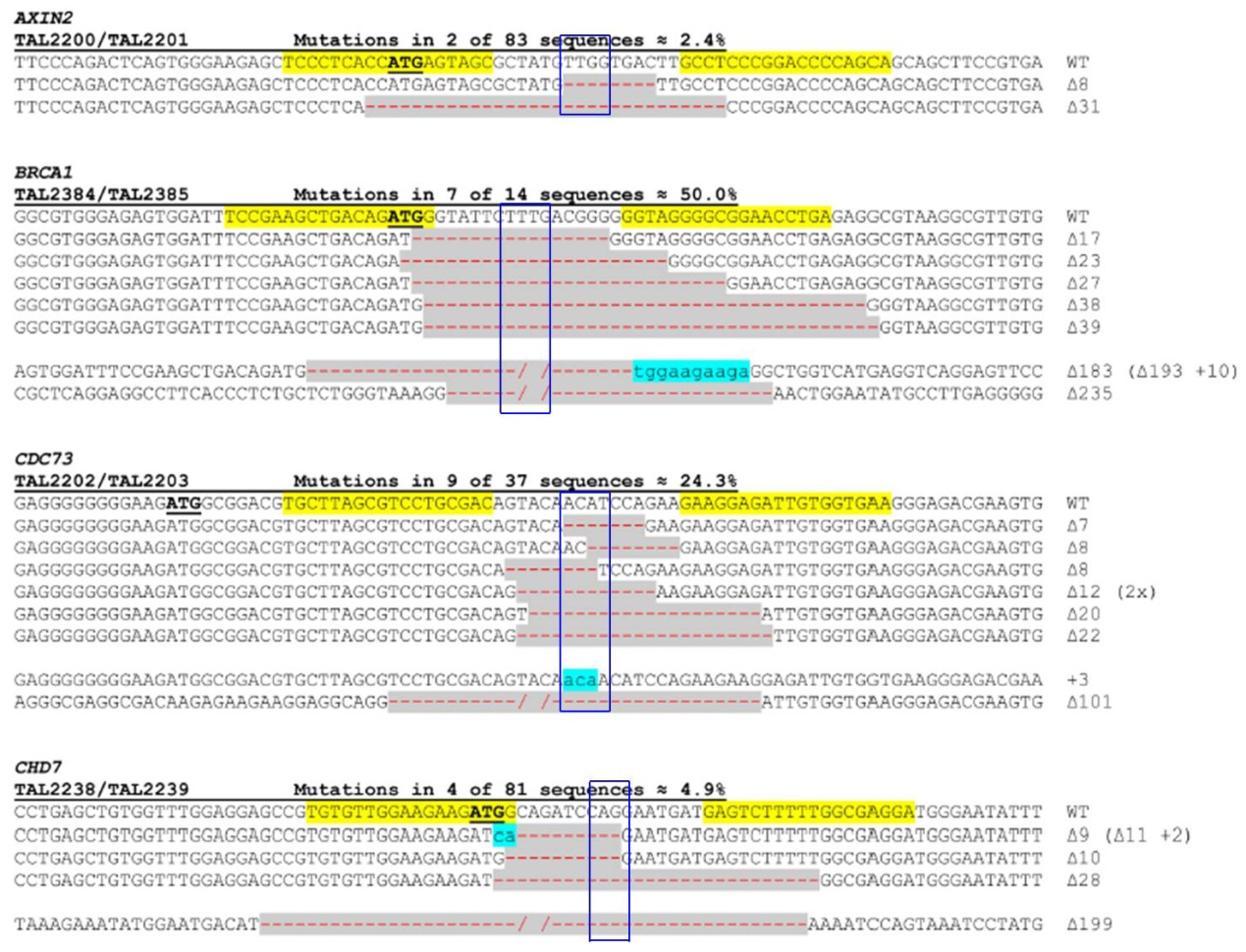


Mutation sequences:21

Mutations including 4 or 5 bases: 16

Mutations including 3 bases: 4

Mutations including 2 bases: 1

Mutations including 1 base: 0


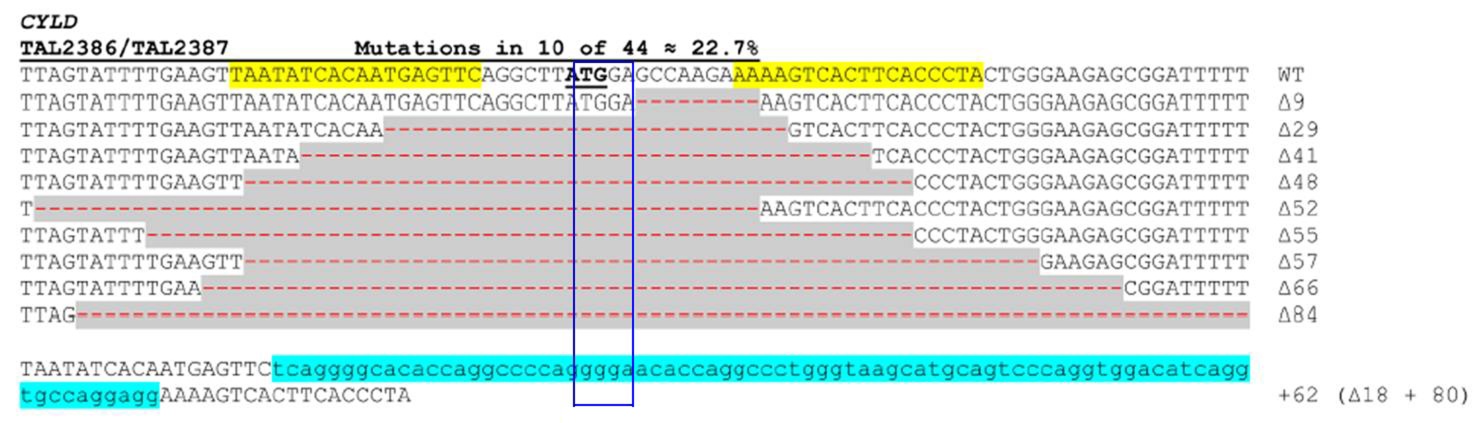


Mutation sequences:10

Mutations including 4 or 5 bases: 9

Mutations including 3 bases: 0

Mutations including 2 bases: 0

Mutations including 1 base: 0


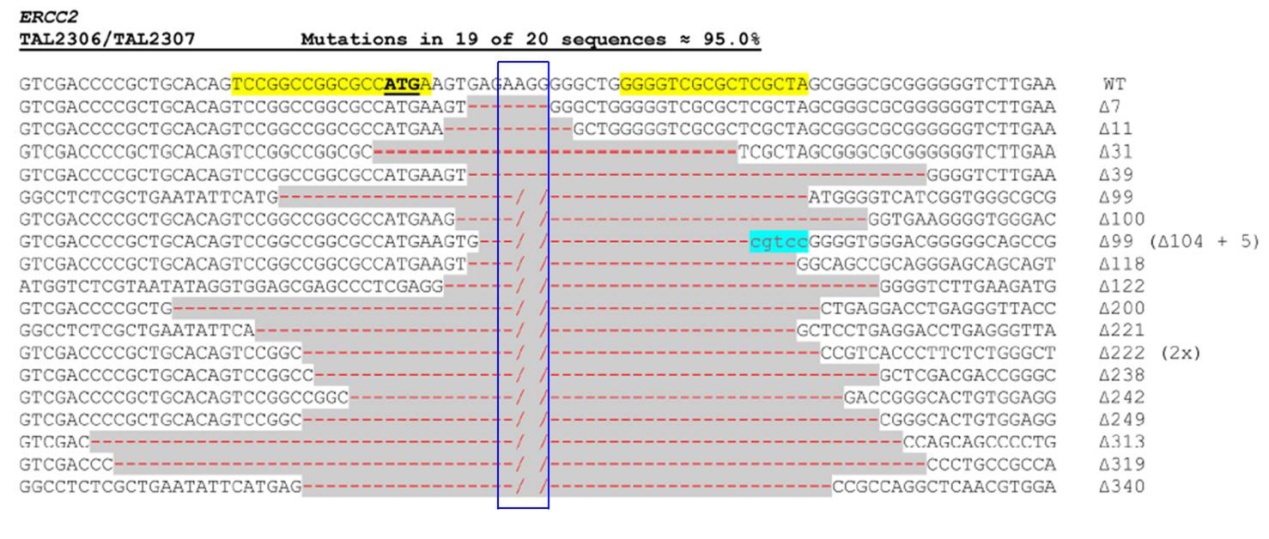


Mutation sequences:19

Mutations including 4 or 5 bases: 19

Mutations including 3 bases: 0

Mutations including 2 bases: 0

Mutations including 1 base: 0


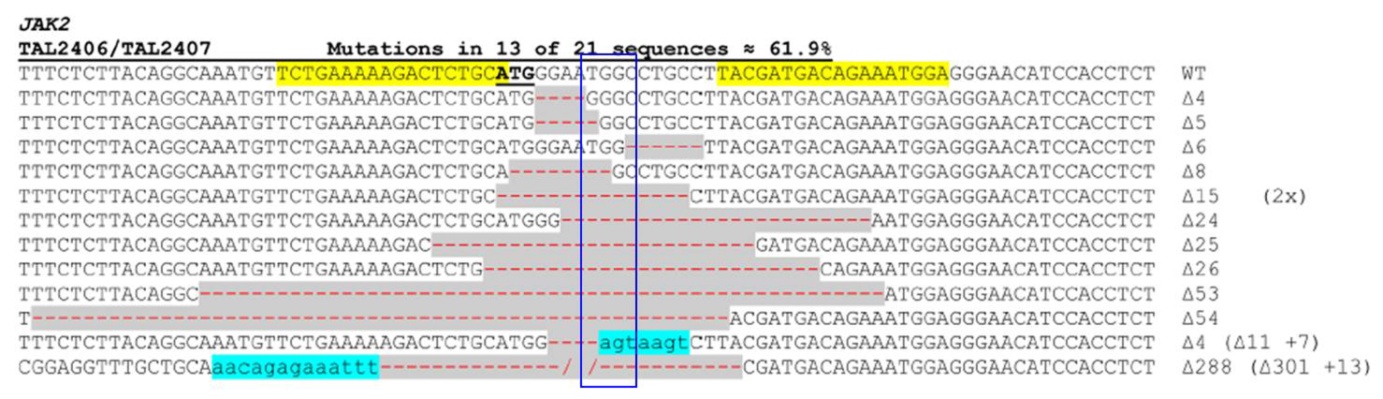


Mutation sequences:13

Mutations including 4 or 5 bases: 9

Mutations including 3 bases: 0

Mutations including 2 bases: 1

Mutations including 1 base: 2


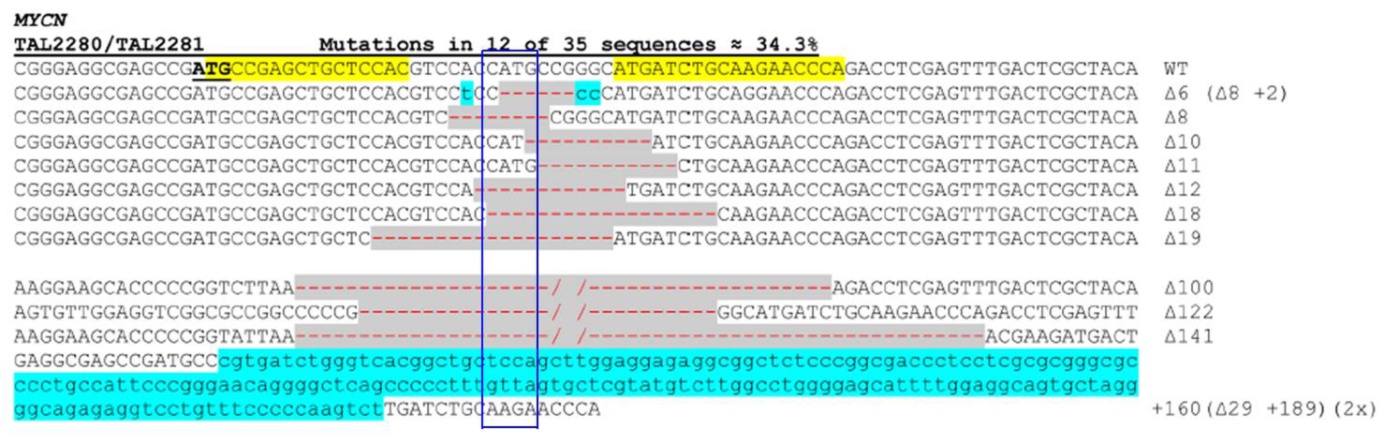


Mutation sequences:12

Mutations including 4 or 5 bases: 9

Mutations including 3 bases: 1

Mutations including 2 bases: 0

Mutations including 1 base: 1


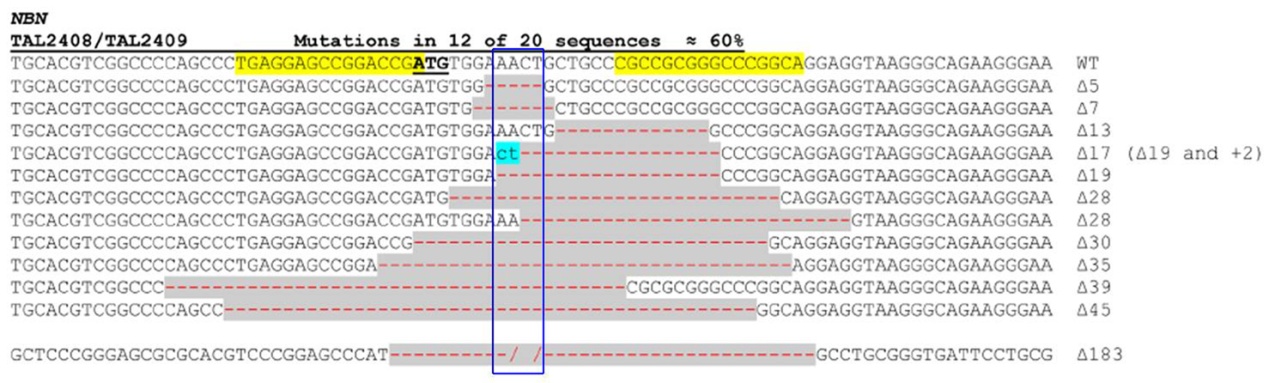


Mutation sequences:12

Mutations including 4 or 5 bases: 10

Mutations including 3 bases: 0

Mutations including 2 bases: 1

Mutations including 1 base: 0


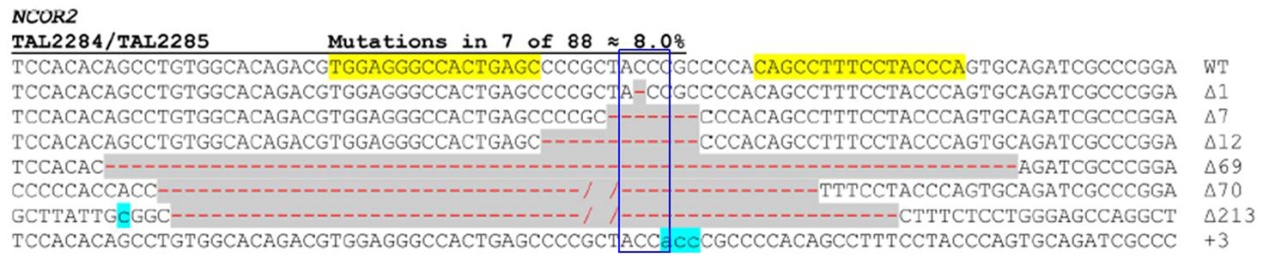


Mutation sequences:7

Mutations including 4 or 5 bases: 5

Mutations including 3 bases: 0

Mutations including 2 bases: 0

Mutations including 1 base: 2


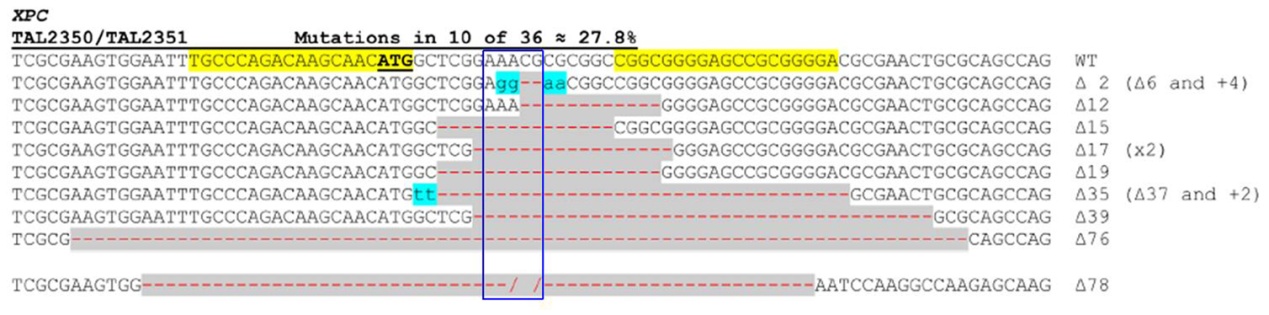


Mutation sequences: 9

Mutations including 4 or 5 bases: 8

Mutations including 3 bases: 1

Mutations including 2 bases: 0

Mutations including 1 base: 0

**Reference 4:** Sander JD, Cade L, Khayter C, *et al*.Targeted gene disruption in somatic zebrafish cells using engineered TALENs. Nature biotechnology, 29(8): 697–698.

**
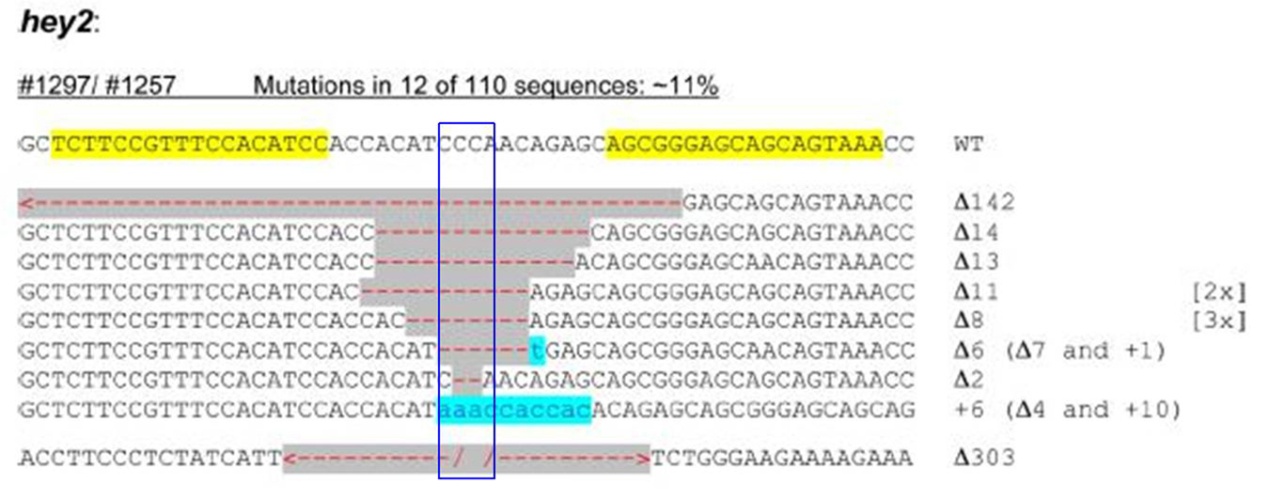
**

Mutation sequences: 12

Mutations including 4 or 5 bases: 11

Mutations including 3 bases: 0

Mutations including 2 bases: 1

Mutations including 1 base: 0


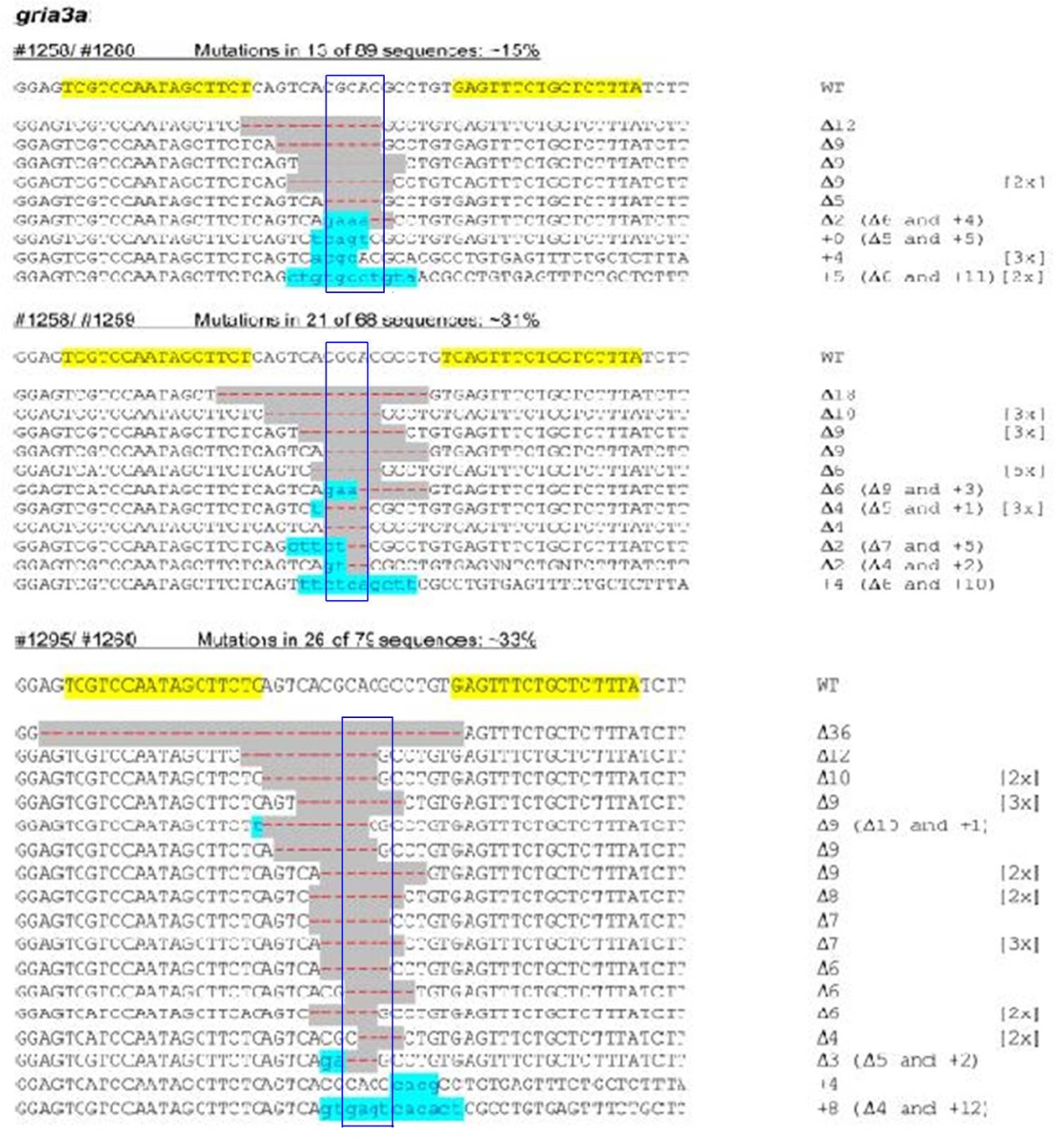


Mutation sequences: 60

Mutations including 4 or 5 bases: 46

Mutations including 3 bases: 12

Mutations including 2 bases: 1

Mutations including 1 base: 0

**Reference 5:** Cade L, Deepak Reyon D, Hwang WY, *et al*. (2012) Highly efficient generation of heritable zebrafish gene mutations using homo- and heterodimeric TALENs. Nucleic Acids Research, 40(16): 8001–8010.


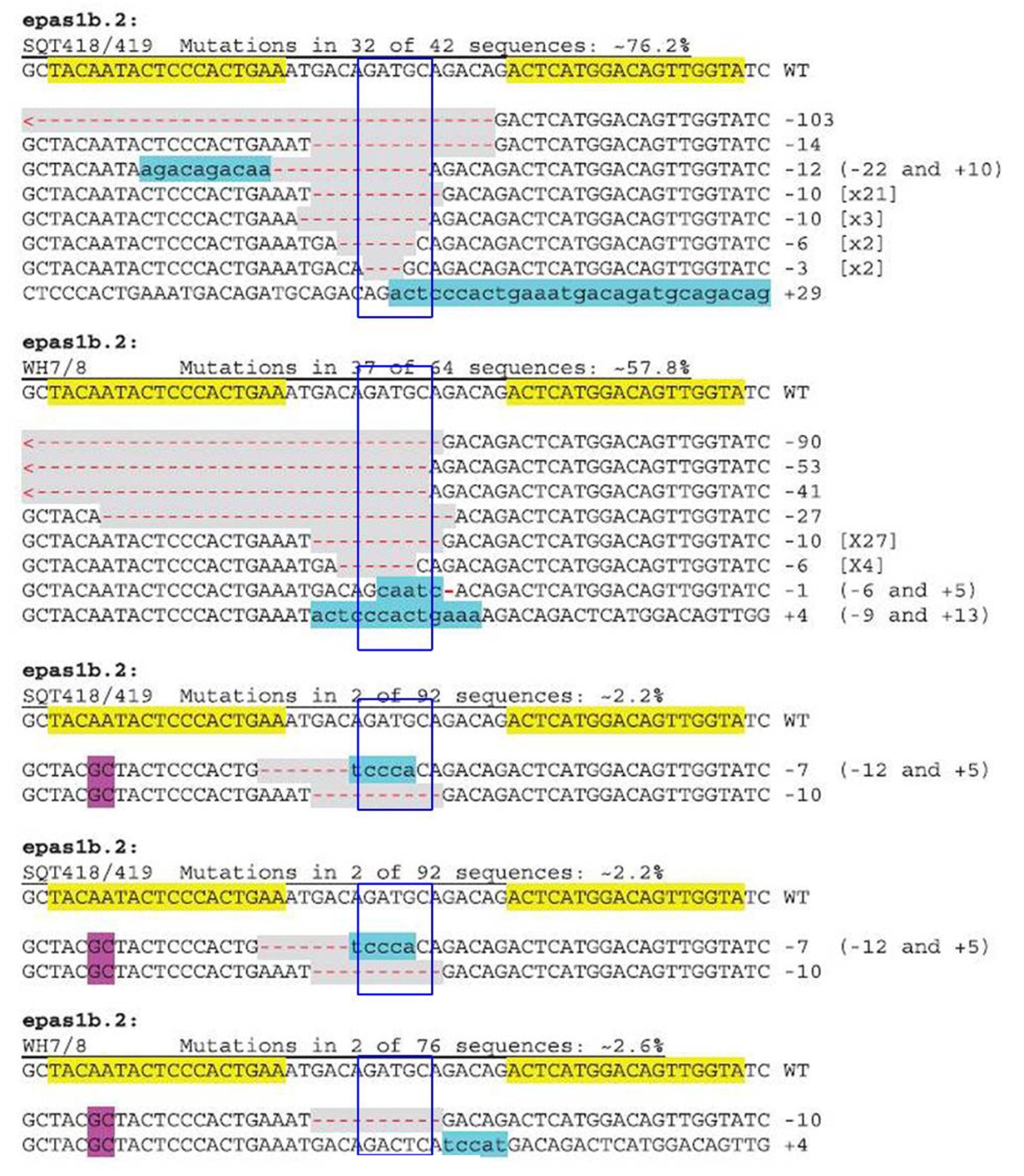


Mutation sequences: 75

Mutations including 4 or 5 bases: 71

Mutations including 3 bases: 3

Mutations including 2 bases: 0

Mutations including 1 base: 0


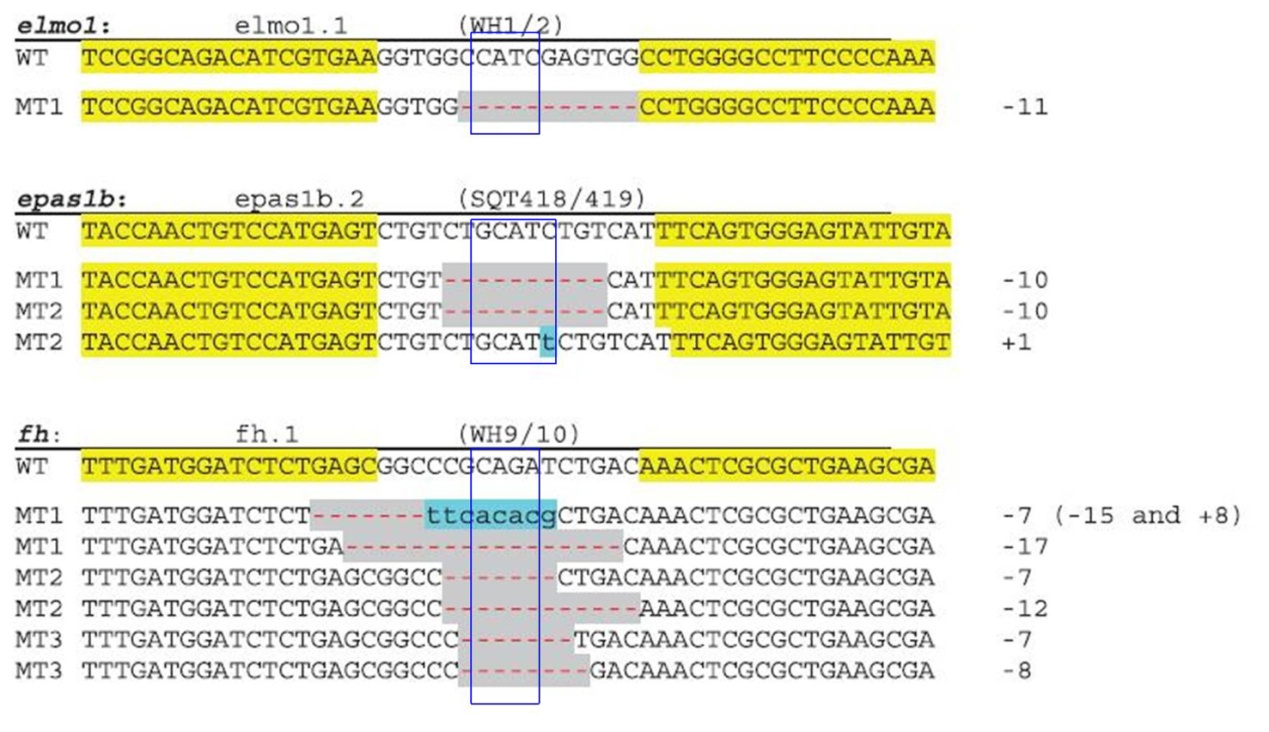


Mutation sequences: 10

Mutations including 4 or 5 bases: 9

Mutations including 3 bases: 0

Mutations including 2 bases: 0

Mutations including 1 base: 1


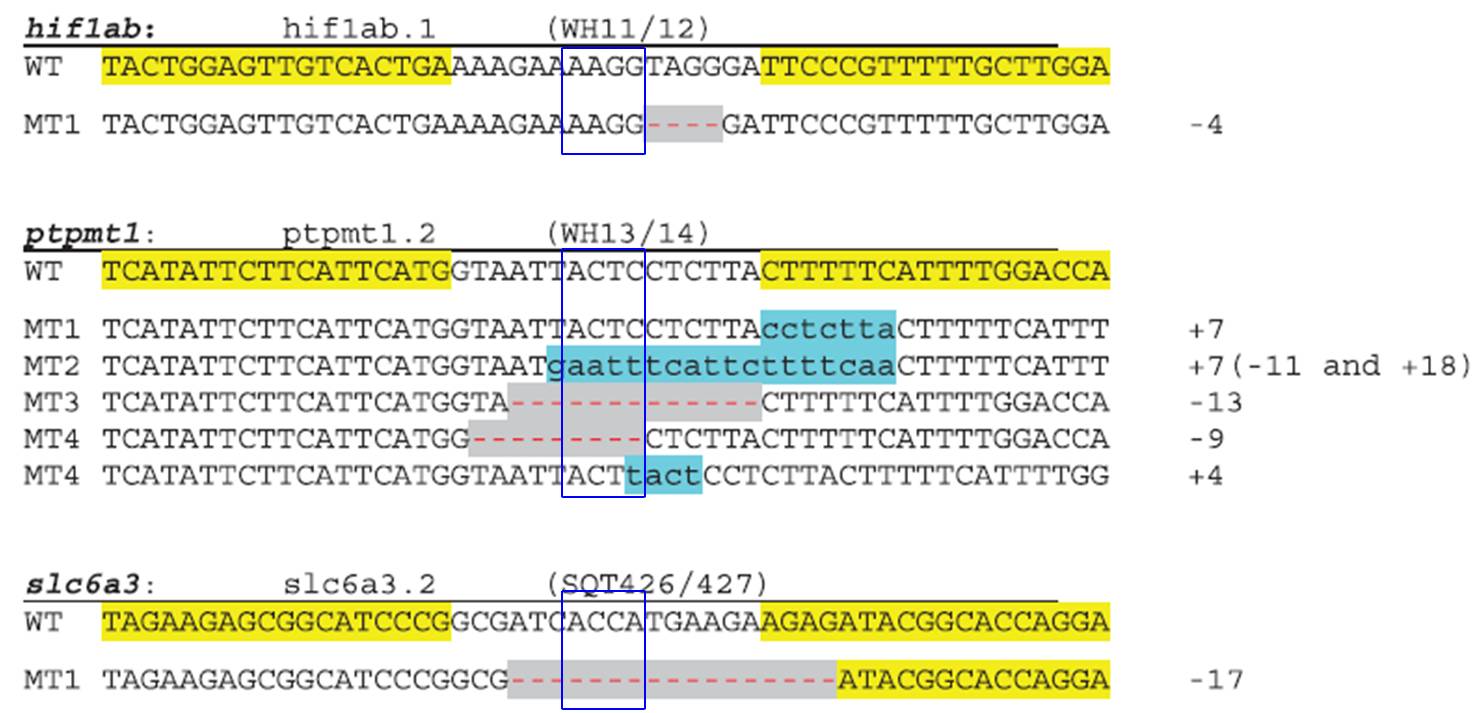


Mutation sequences: 7

Mutations including 4 or 5 bases: 4

Mutations including 3 bases: 0

Mutations including 2 bases: 0

Mutations including 1 base: 1

**Reference 6:** Cermak T, Erin L. Doyle E, Christian M, *et al*. (2011) Efficient design and assembly of custom TALEN and other TAL effector-based constructs for DNA targeting. Nucleic Acids Research, 39(12): e82.


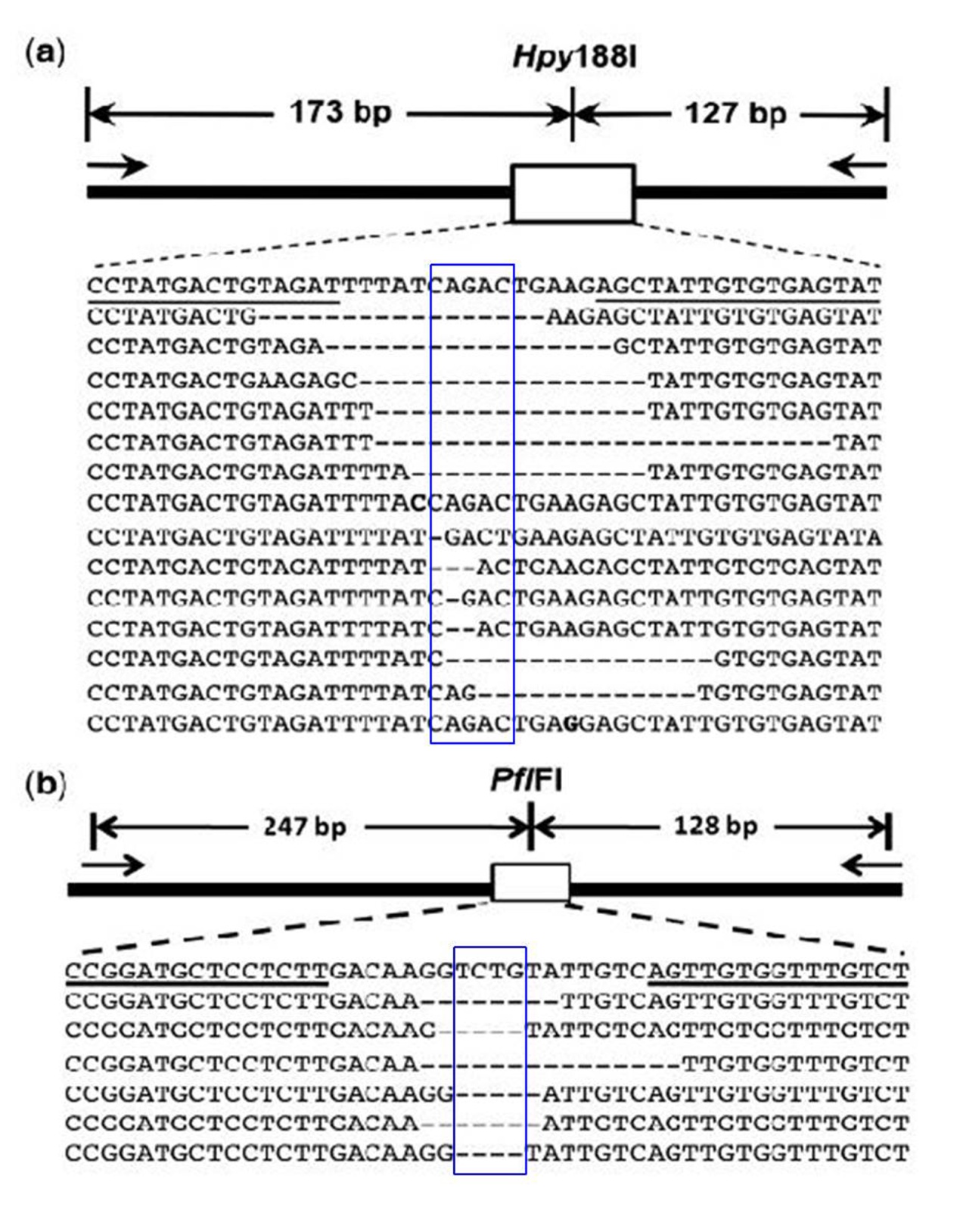


Mutation sequences: 20

Mutations including 4 or 5 bases: 13

Mutations including 3 bases: 1

Mutations including 2 bases: 2

Mutations including 1 base: 2

**Reference 7:** Li T, Huang S, Zhao X, *et al*. (2011)Modularly assembled designer TAL effector nucleases for targeted gene knockout and gene replacement in eukaryotes. Nucleic Acids Research, 39(14): 6315–6325.


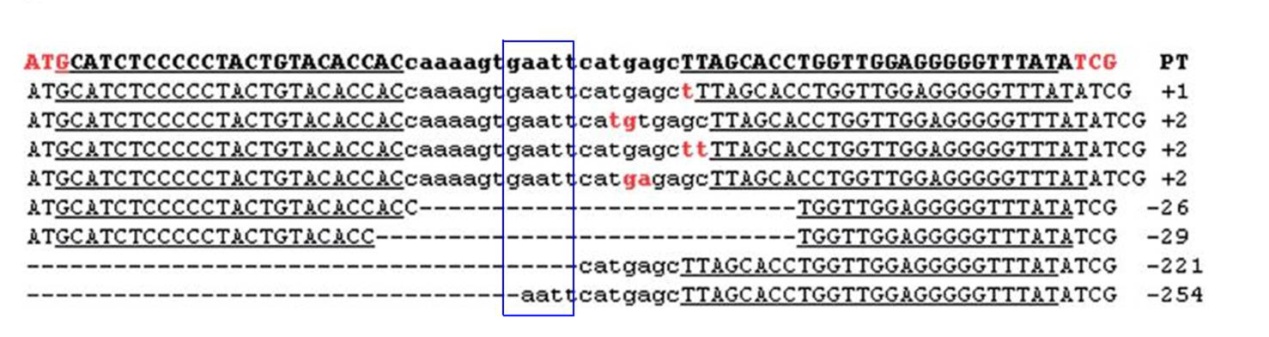


Mutation sequences: 8

Mutations including 4 or 5 bases: 3

Mutations including 3 bases: 0

Mutations including 2 bases: 0

Mutations including 1 base: 1


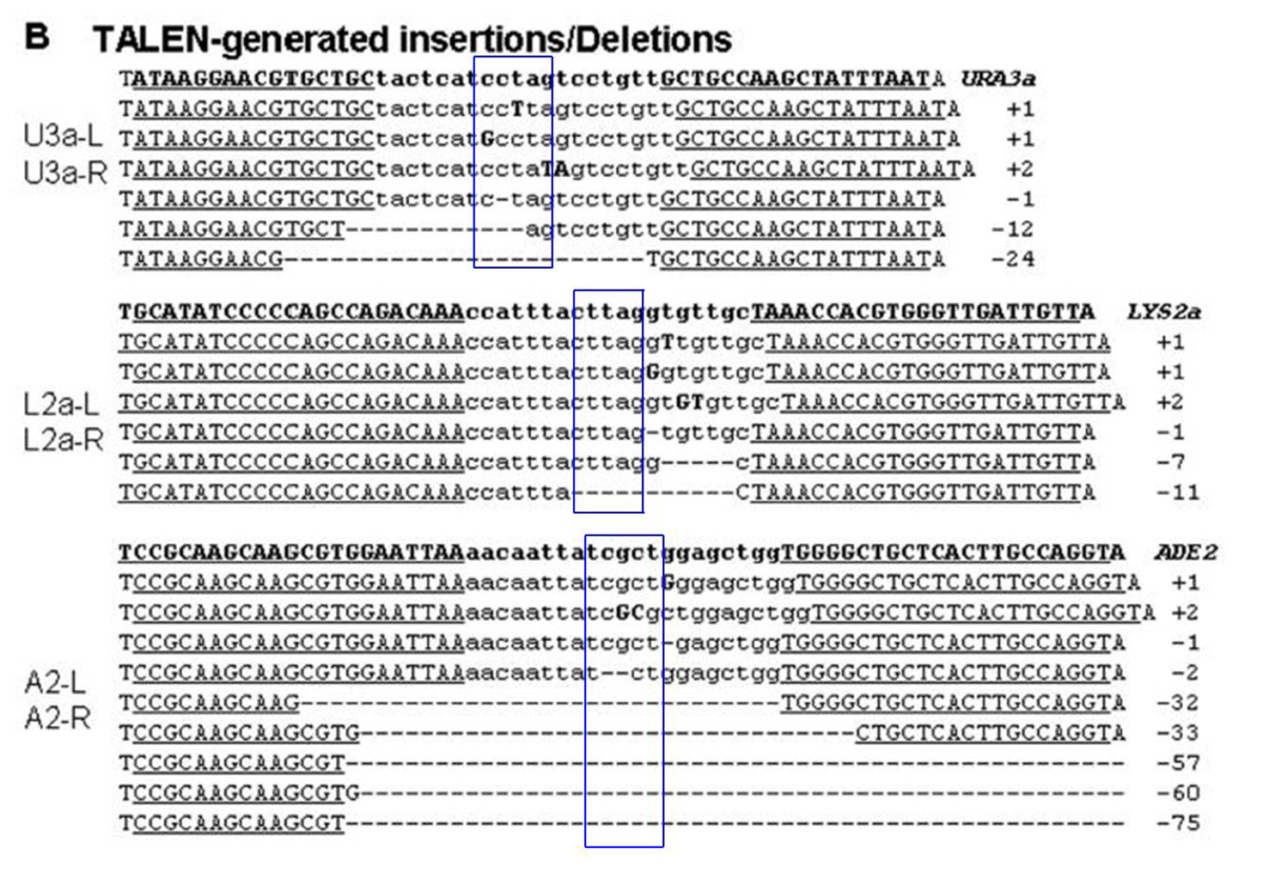


Mutation sequences: 21

Mutations including 4 or 5 bases: 7

Mutations including 3 bases: 1

Mutations including 2 bases: 2

Mutations including 1 base: 4

**Reference 8:** Miller JC, Tan S, Qiao G, *et al*. (2011) A TALE nuclease architecture for efficient genome editing. Nature biotechnology, 29(2): 145-148.


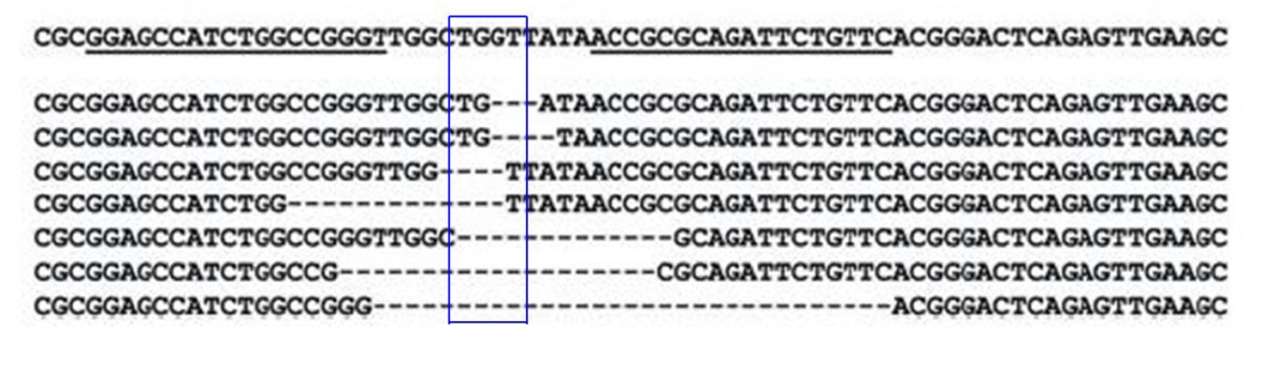


Mutation sequences: 7

Mutations including 4 or 5 bases: 3

Mutations including 3 bases: 2

Mutations including 2 bases: 2

Mutations including 1 base: 0

**Reference 9:** Carlson DF, Tan W, Lillico SG, *et al*. (2012) Efficient TALEN-mediated gene knockout in livestock. PNAS, 109 (43): 17382–17387.


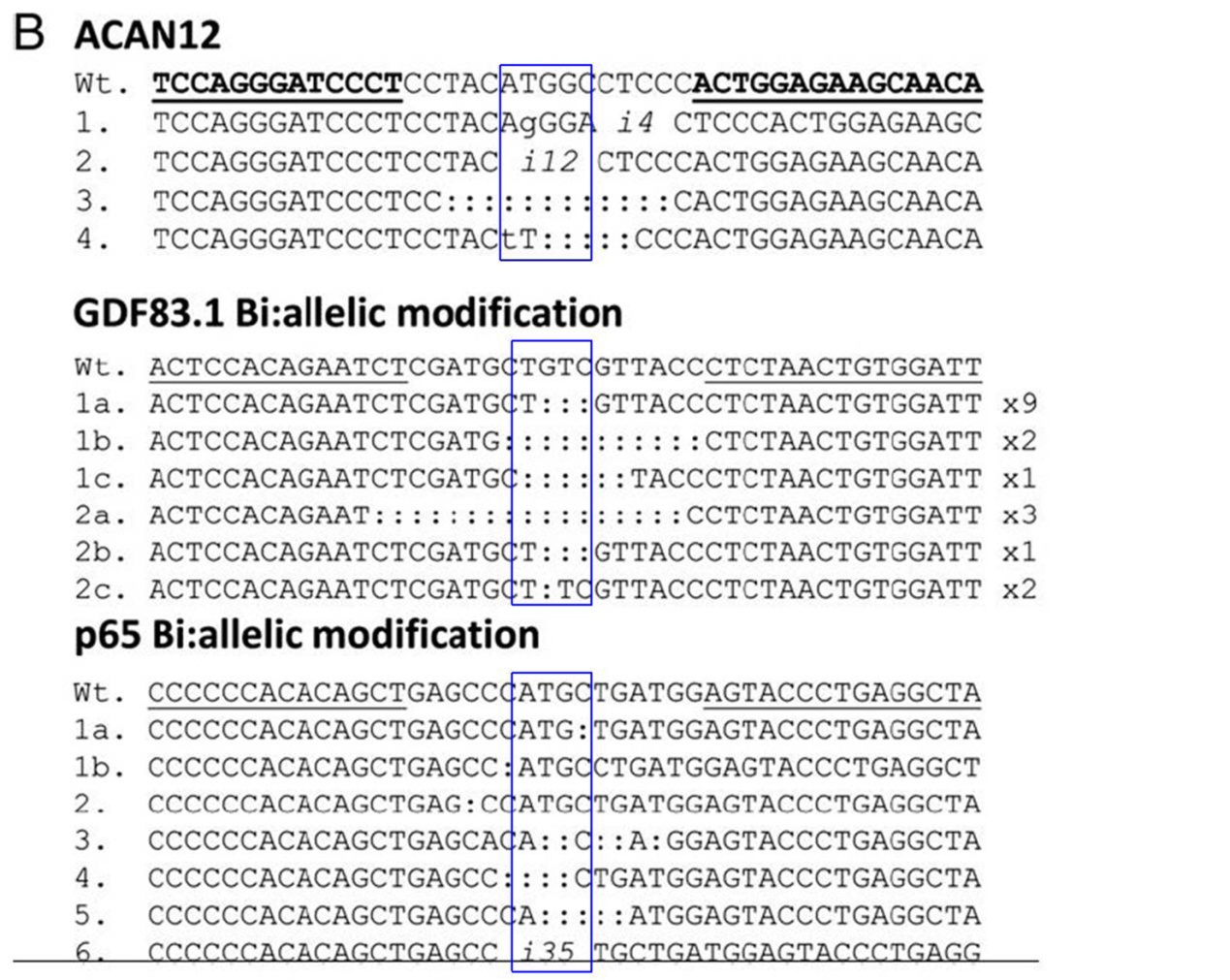


Mutation sequences: 17

Mutations including 4 or 5 bases: 6

Mutations including 3 bases: 5

Mutations including 2 bases: 2

Mutations including 1 base: 2


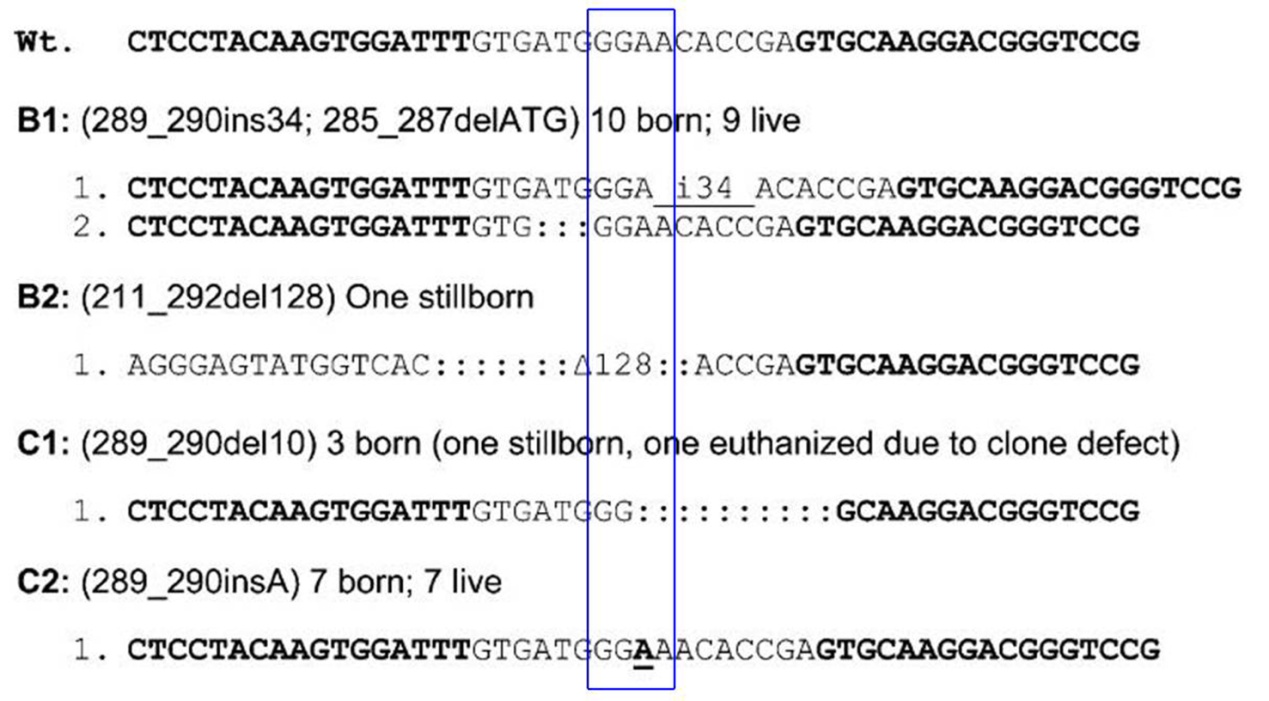


Mutation sequences: 5

Mutations including 4 or 5 bases: 1

Mutations including 3 bases: 0

Mutations including 2 bases: 1

Mutations including 1 base: 2

**Reference 10:** Ma S, Zhang S, Wang F, *et al*. (2012) Highly Efficient and Specific Genome Editing in Silkworm Using Custom TALENs. PLOS ONE, 7(9):e45035.


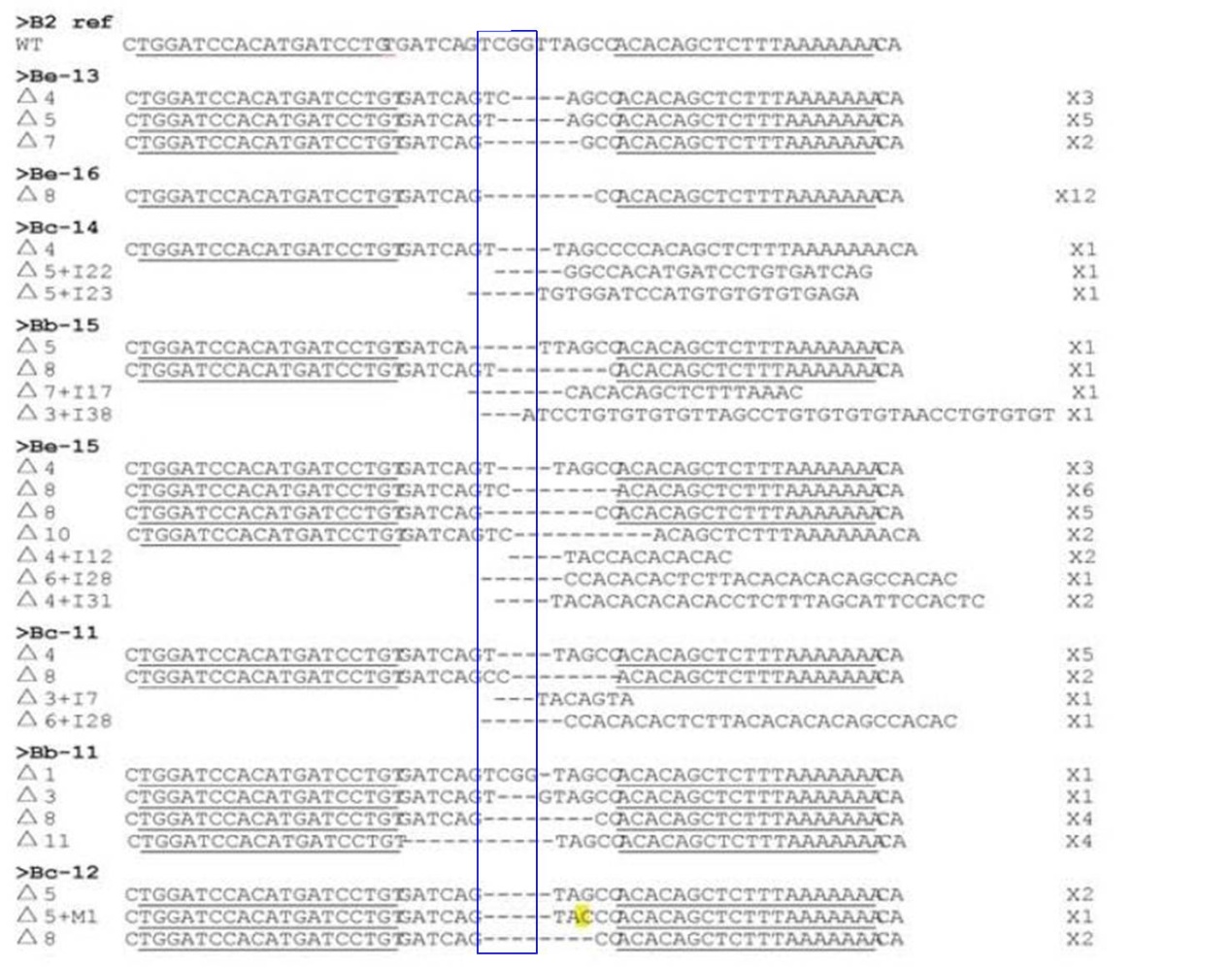


Mutation sequences: 74

Mutations including 4 or 5 bases: 42

Mutations including 3 bases: 17

Mutations including 2 bases: 14

Mutations including 1 base: 0


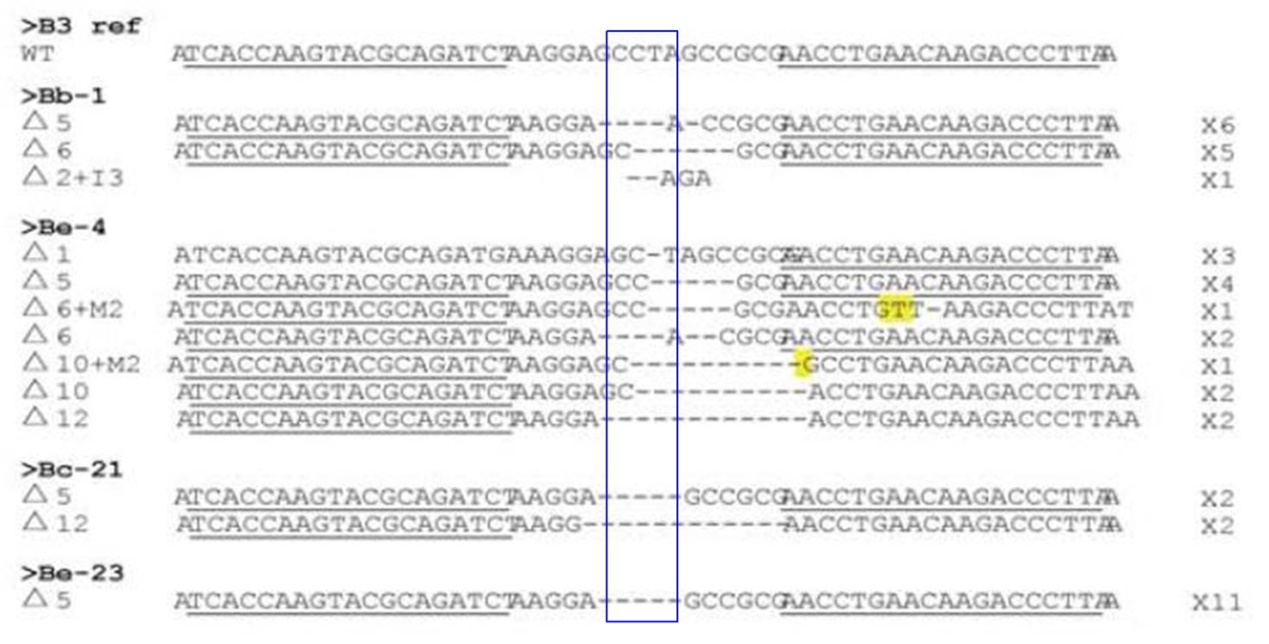


Mutation sequences: 42

Mutations including 4 or 5 bases: 17

Mutations including 3 bases: 17

Mutations including 2 bases: 5

Mutations including 1 base: 3

**Reference 11:** Sung YH, Baek IJ, Kim DH, *et al*. (2013) Knockout mice created by TALENmediated gene targeting. Nature biotechnology, 31(1): 23–24.


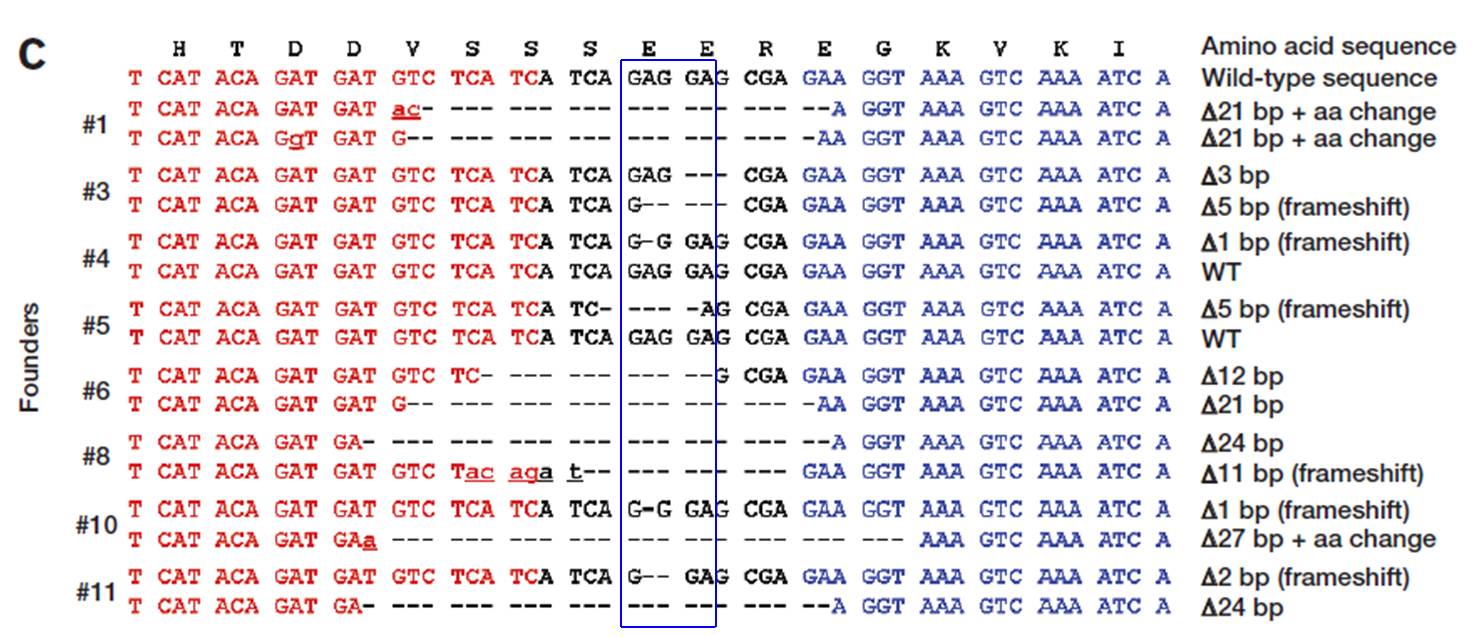


Mutation sequences: 16

Mutations including 4 or 5 bases: 10

Mutations including 3 bases: 0

Mutations including 2 bases: 2

Mutations including 1 base: 2

**Reference 12:** Ding Q, Lee YK, Schaefer EAK, *et al*. (2013) A TALEN Genome-Editing System for Generating Human Stem Cell-Based Disease Models. Cell Stem Cell, 12:238–251.


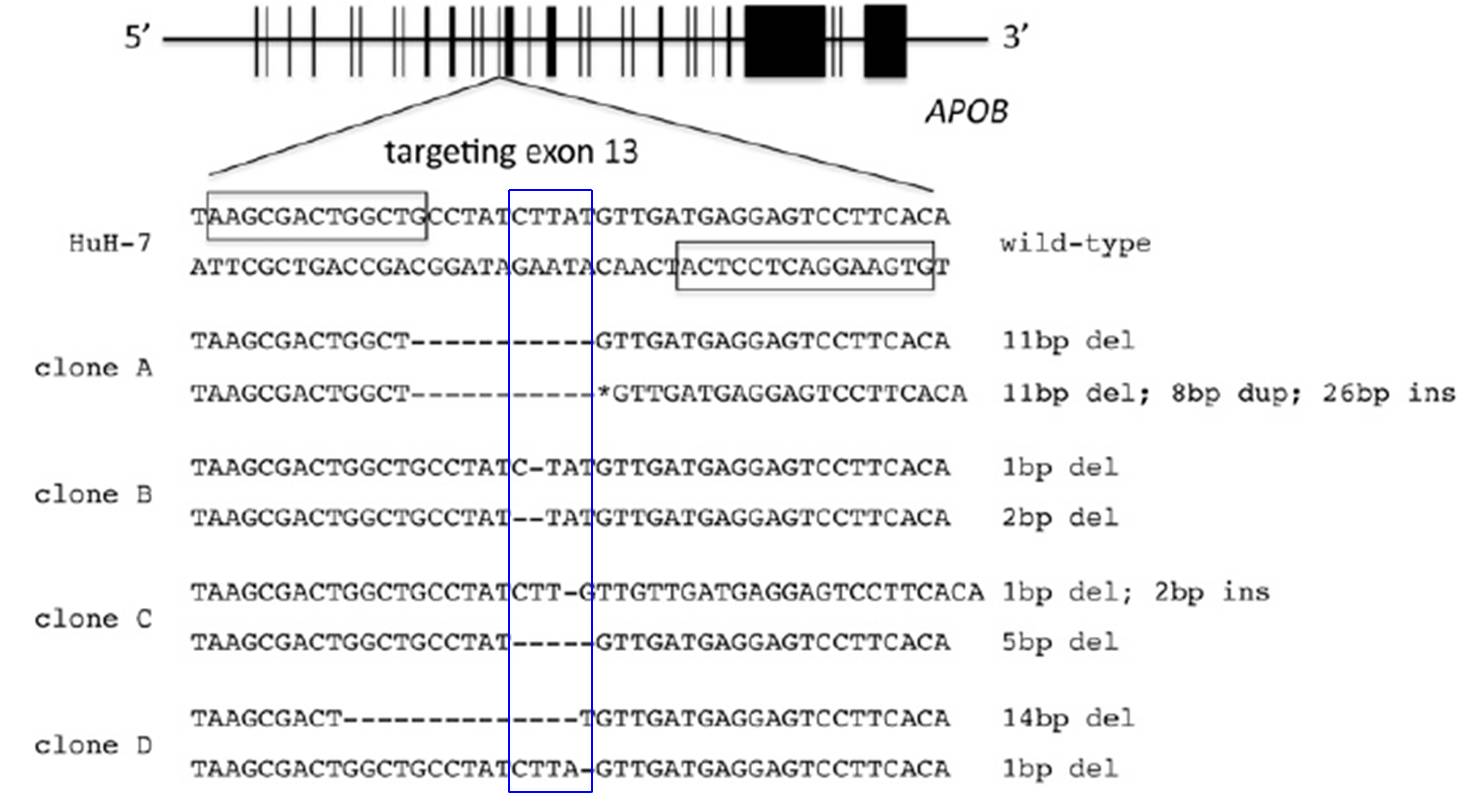


Mutation sequences: 8

Mutations including 4 or 5 bases: 4

Mutations including 3 bases: 0

Mutations including 2 bases: 1

Mutations including 1 base: 3


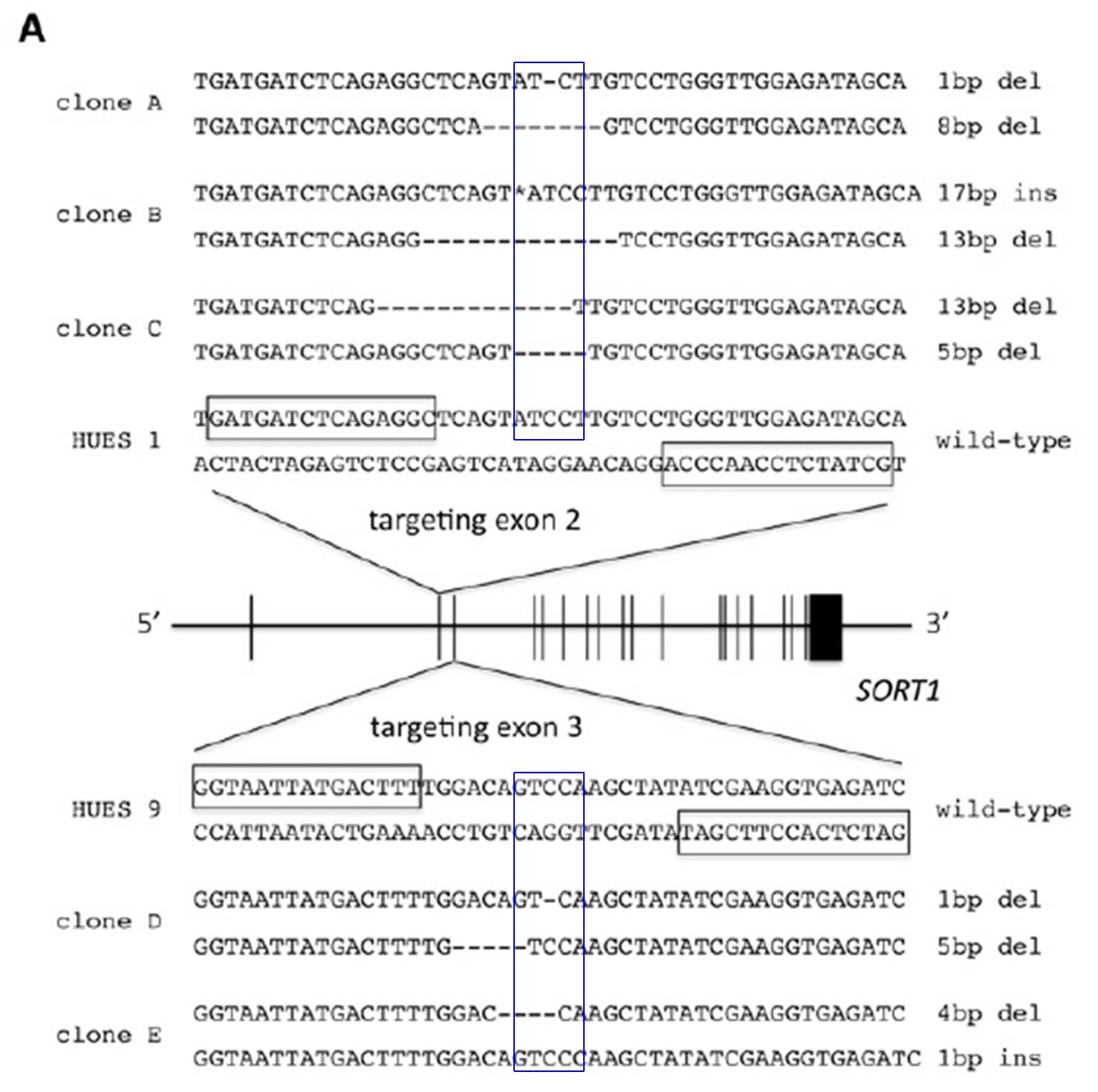


Mutation sequences: 10

Mutations including 4 or 5 bases: 4

Mutations including 3 bases: 1

Mutations including 2 bases: 0

Mutations including 1 base: 5


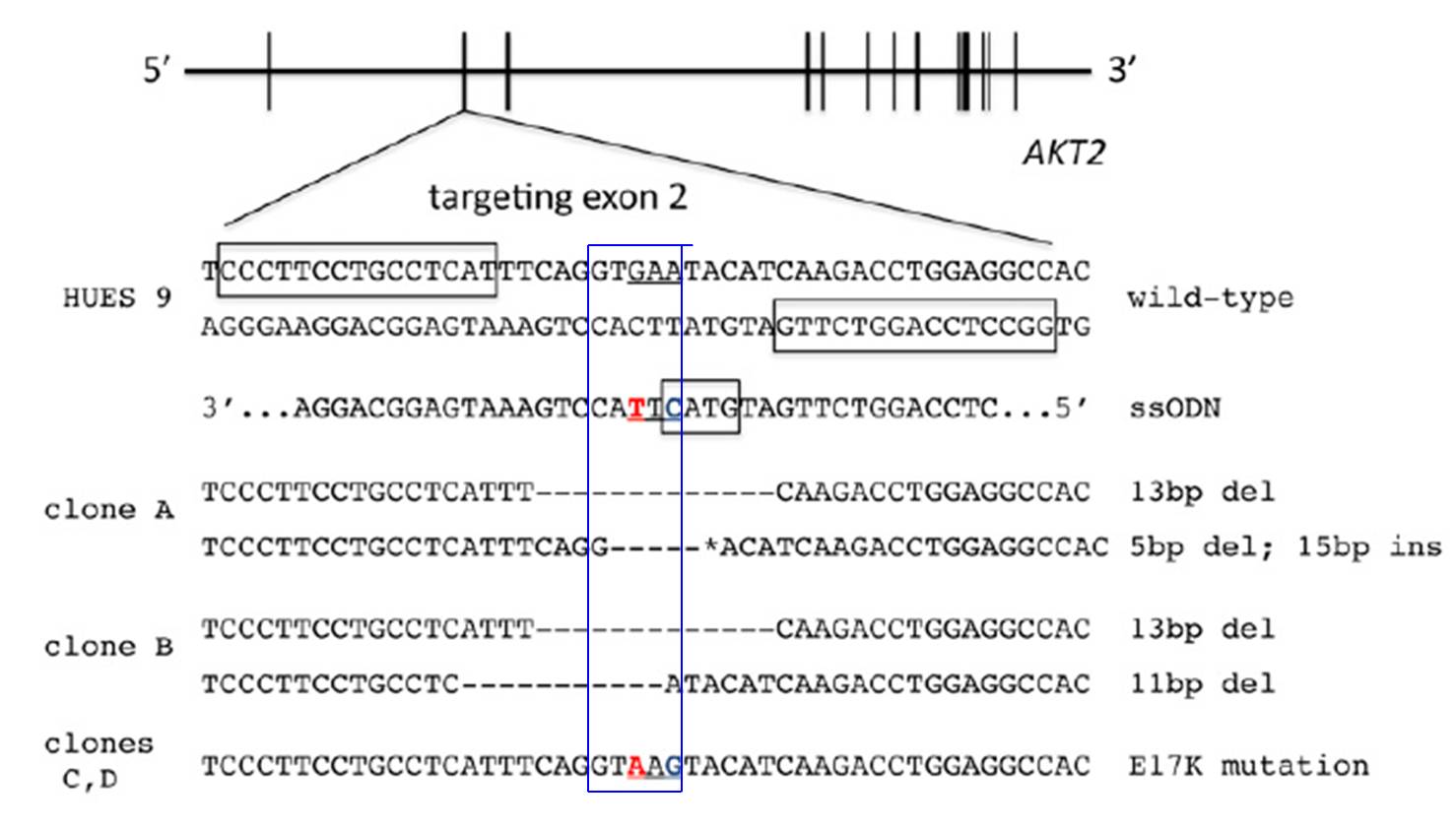


Mutation sequences: 6

Mutations including 4 or 5 bases: 4

Mutations including 3 bases: 0

Mutations including 2 bases: 2

Mutations including 1 base: 0


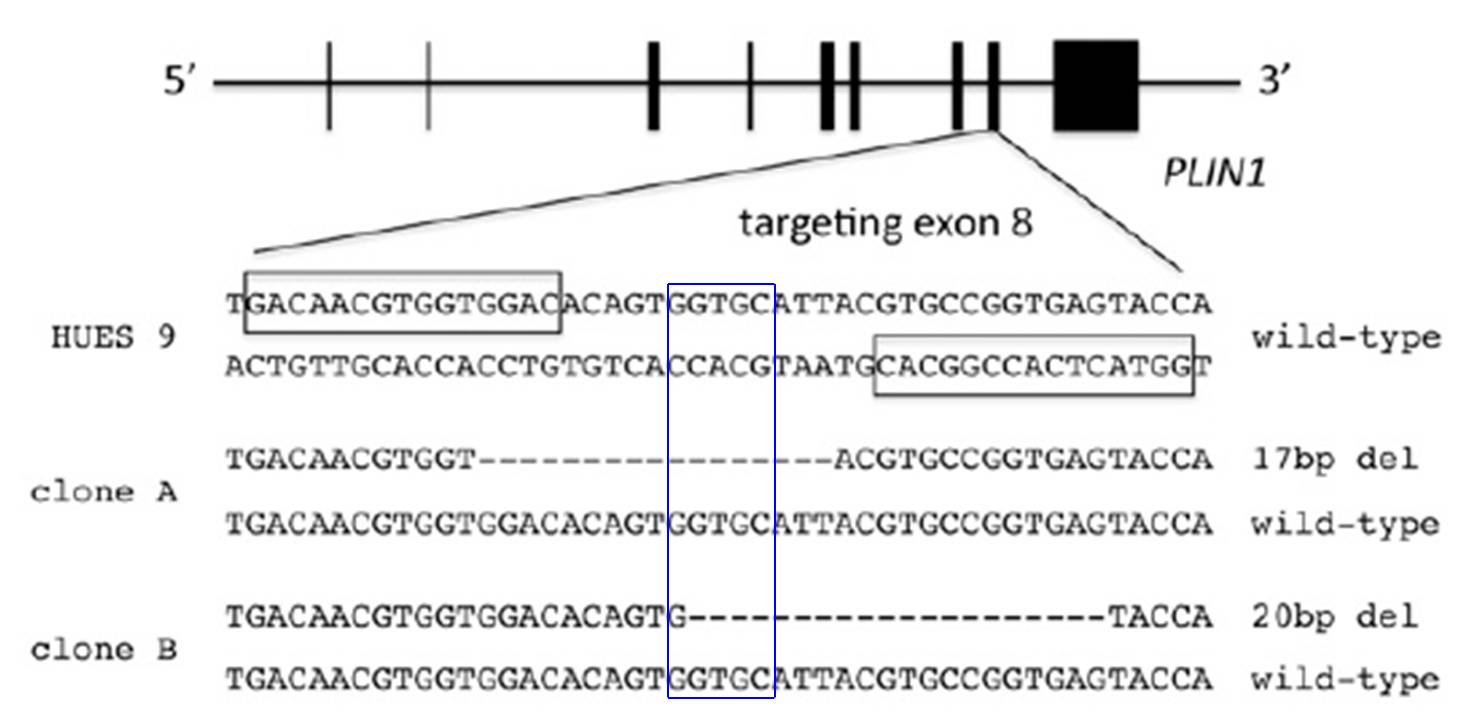


Mutation sequences: 2

Mutations including 4 or 5 bases: 2

Mutations including 3 bases: 0

Mutations including 2 bases: 0

Mutations including 1 base: 0

**Table 1**. TALENs induced mutant sequence statistics

|  | Mutation type | Mutant sequence number | rato |
| --- | --- | --- | --- |
| Mutational hot spot regions (MHS): the middle 4-5 base pairs of the spacer regions | Mutant sites (4 or 5 bases) | 459 | 70.18% |
|  | Mutant sites (3 bases) | 75 | 11.47% |
|  | Mutant sites (2 bases) | 48 | 7.34% |
|  | Mutant sites (1 bases) | 43 | 6.57% |
|  | All mutants at MHS | 625 | 95.57% |
|  | Total mutant sequences | 654 |  |
