# Supplementary material for: A PCR Based Protocol for Detecting Indel Mutations Induced by TALENs and CRISPR/Cas9 in Zebrafish
Source: PLoS One. 2014 Jun 5;9(6):e98282. doi: 10.1371/journal.pone.0098282 (PMC4046980; doi:10.1371/journal.pone.0098282)
Supplement: Document S2 — Sequence analysis of Cas9 induced mutations. (DOCX) [file pone.0098282.s009.docx]

**Sequence analysis of Cas9 induced mutations**

目录

[**Reference 1** 2](#_Toc385581224)

[**Reference 2**. 3](#_Toc385581225)

[**Reference 3**. 3](#_Toc385581226)

[Supplementary Materials 4](#_Toc385581227)

[**Reference 4**. 4](#_Toc385581228)

[Supplementary Materials 5](#_Toc385581229)

[**Reference 5**. 7](#_Toc385581230)

[**Reference 6**. 8](#_Toc385581231)

[**Reference 7**. 9](#_Toc385581232)

[**Reference 8** 11](#_Toc385581233)

[**Reference 9** 11](#_Toc385581234)

[Supplementary Materials 12](#_Toc385581235)

[**Table 1** 15](#_Toc385581236)

**Reference 1:** Wang H, Hui Yang H, Shivalila CS, *et al*. (2013)One-Step Generation of Mice Carrying Mutations in Multiple Genes by CRISPR/Cas-Mediated Genome Engineering. Cell, 153: 910–918.


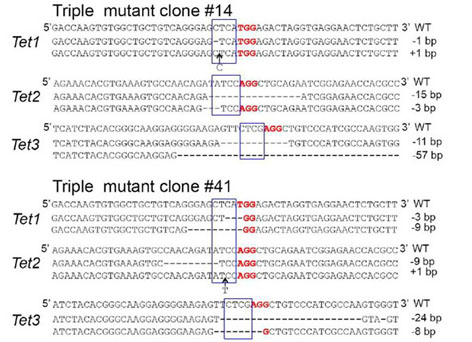


Mutation sequences: 12

Mutations including 4 bases: 6

Mutations including 3 bases: 0

Mutations including 2 bases: 1

Mutations including 1 base: 5


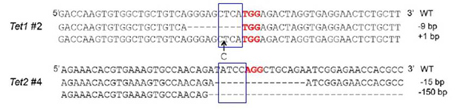


Mutation sequences: 4

Mutations including 4 bases: 3

Mutations including 3 bases: 0

Mutations including 2 bases: 0

Mutations including 1 base: 1


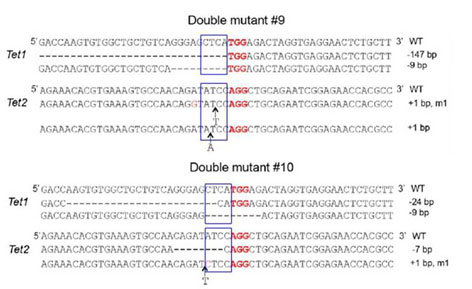


Mutation sequences: 8

Mutations including 4 bases: 3

Mutations including 3 bases: 1

Mutations including 2 bases: 1

Mutations including 1 base: 3

**Reference 2:** Cong L, Ran FA, Cox D, *et al*. (2013) Multiplex Genome Engineering Using CRISPR/Cas Systems. Scienceexpress reports. 1–8.


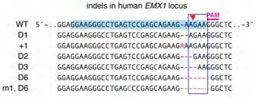


Mutation sequences: 6

Mutations including 4 bases: 2

Mutations including 3 bases: 0

Mutations including 2 bases: 1

Mutations including 1 base: 1

**Reference 3:** Cho SW, Kim S, Kim JM, *et al*. Targeted genome engineering in human cells with RNA-guided endonucleases. Nature Biotechnology, 31(3): 230–232.

**
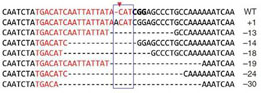
**

Mutation sequences: 7

Mutations including 4 bases: 6

Mutations including 3 bases: 0

Mutations including 2 bases: 0

Mutations including 1 base: 1

**
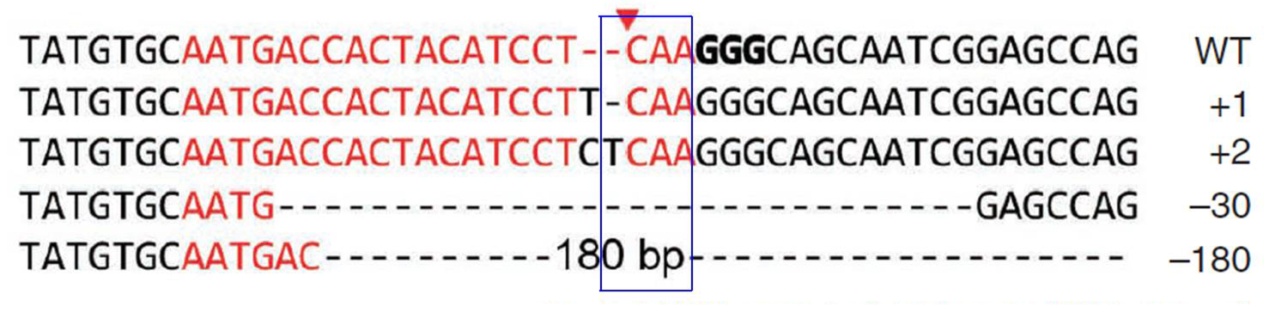
**

Mutation sequences: 4

Mutations including 4 bases: 2

Mutations including 3 bases: 0

Mutations including 2 bases: 0

Mutations including 1 base: 2

**Supplementary Materials**


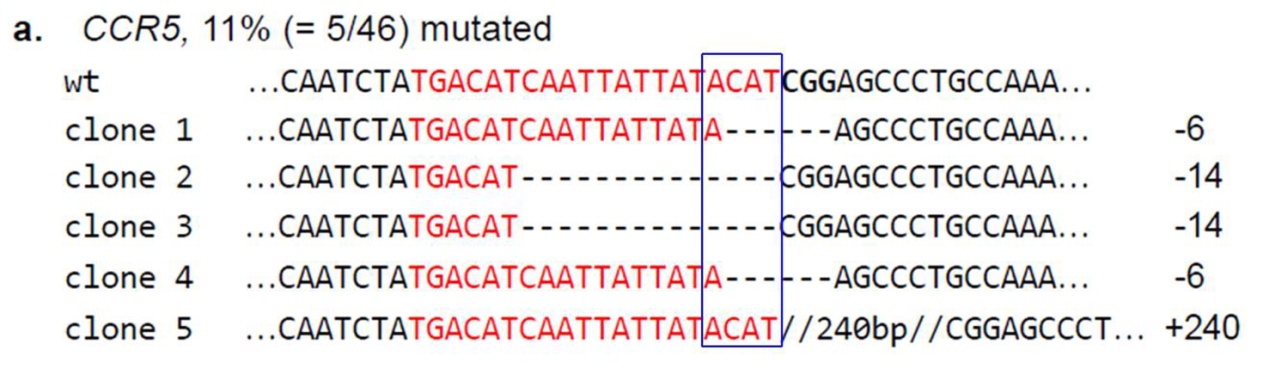


Mutation sequences: 5

Mutations including 4 bases: 2

Mutations including 3 bases: 2

Mutations including 2 bases: 0

Mutations including 1 base: 0

**Reference 4:** Hwang WY, Fu Y, Reyon D, *et al*. (2013) Efficient genome editing in zebrafish using a CRISPR-Cas system. Nature Biotechnology, 31: 227–229.


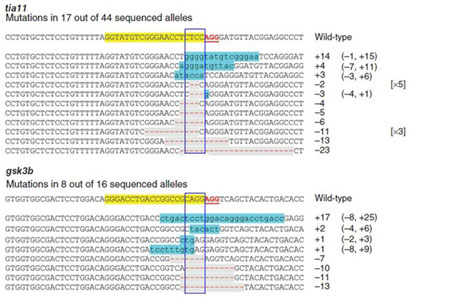


Mutation sequences: 25

Mutations including 4 bases: 12

Mutations including 3 bases: 6

Mutations including 2 bases: 7

Mutations including 1 base: 0

**Supplementary Materials**


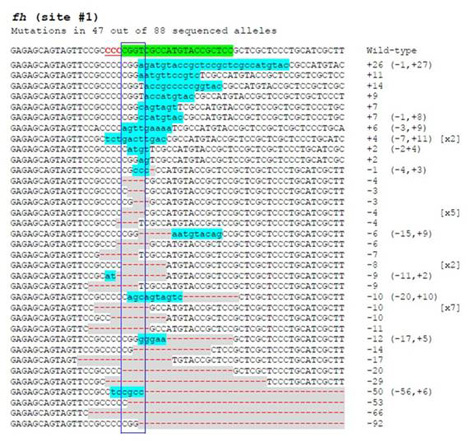


Mutation sequences: 47

Mutations including 4 bases: 27

Mutations including 3 bases: 7

Mutations including 2 bases: 3

Mutations including 1 base: 8


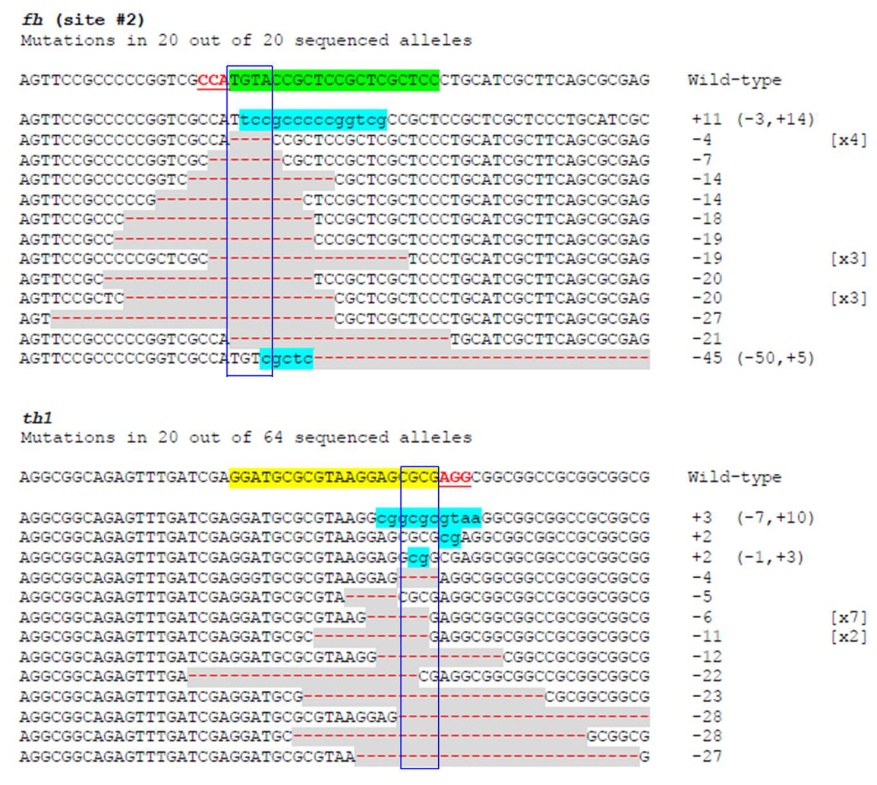


Mutation sequences: 40

Mutations including 4 bases: 25

Mutations including 3 bases: 10

Mutations including 2 bases: 2

Mutations including 1 base: 1


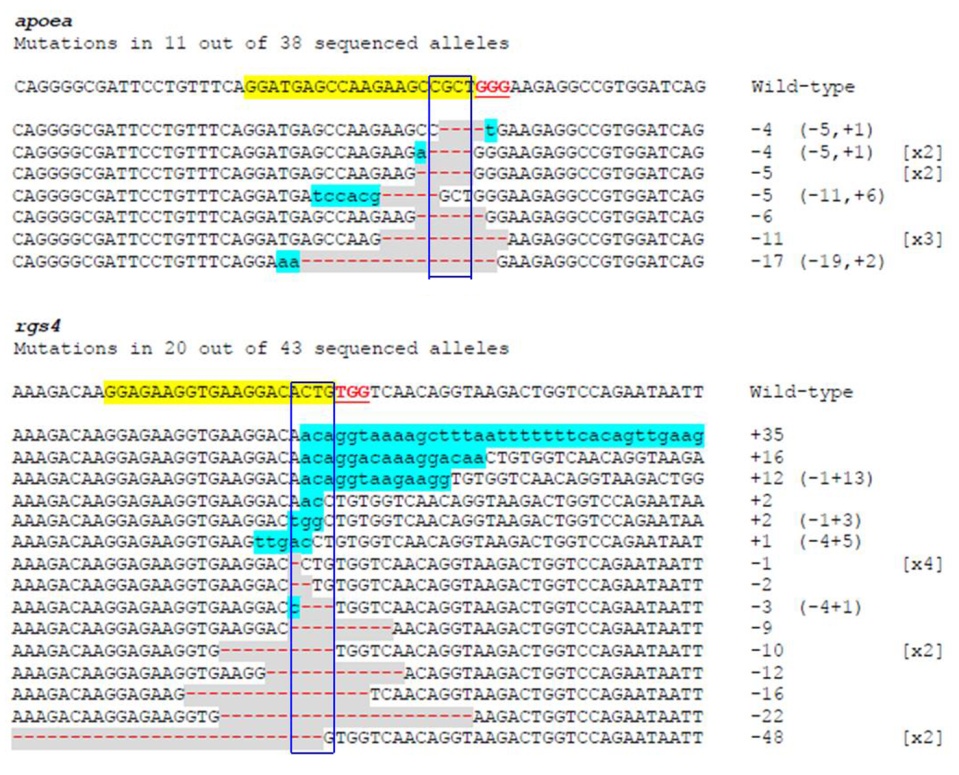


Mutation sequences: 31

Mutations including 4 bases: 17

Mutations including 3 bases: 7

Mutations including 2 bases: 2

Mutations including 1 base: 5


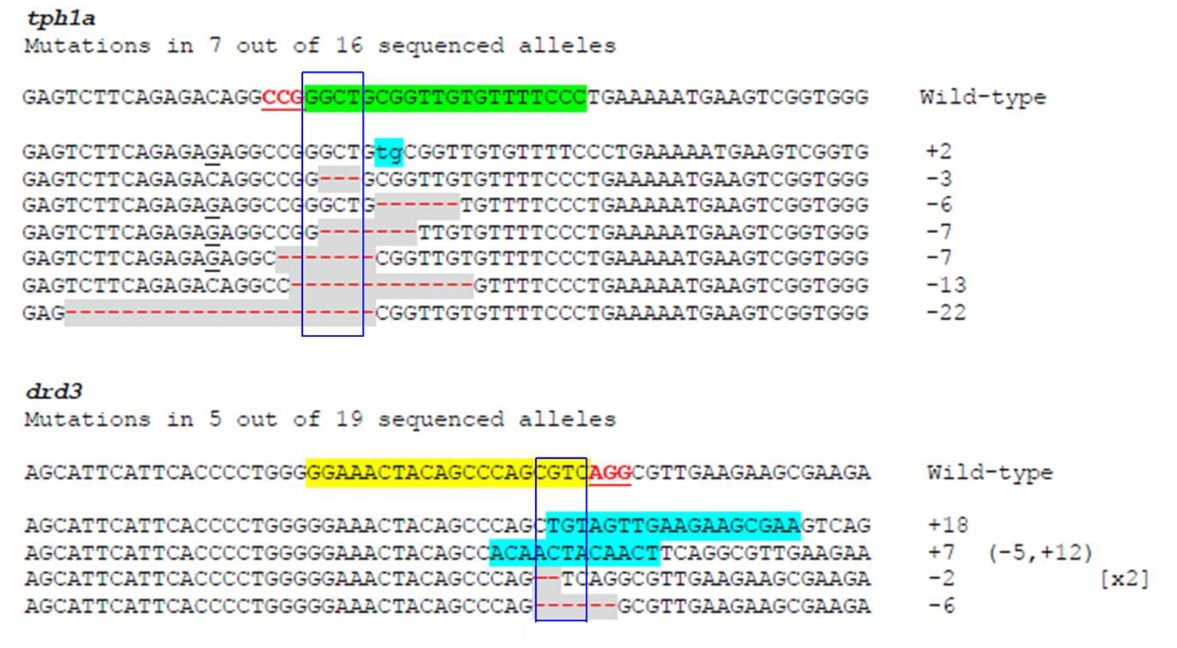


Mutation sequences: 12

Mutations including 4 bases: 5

Mutations including 3 bases: 3

Mutations including 2 bases: 2

Mutations including 1 base: 0

**Reference 5:** Chang N, Sun C, Lu Gao L, *et al*. (2013) Genome editing with RNA-guided Cas9 nuclease in Zebrafish embryos. Cell Research, 23:465-472.

**
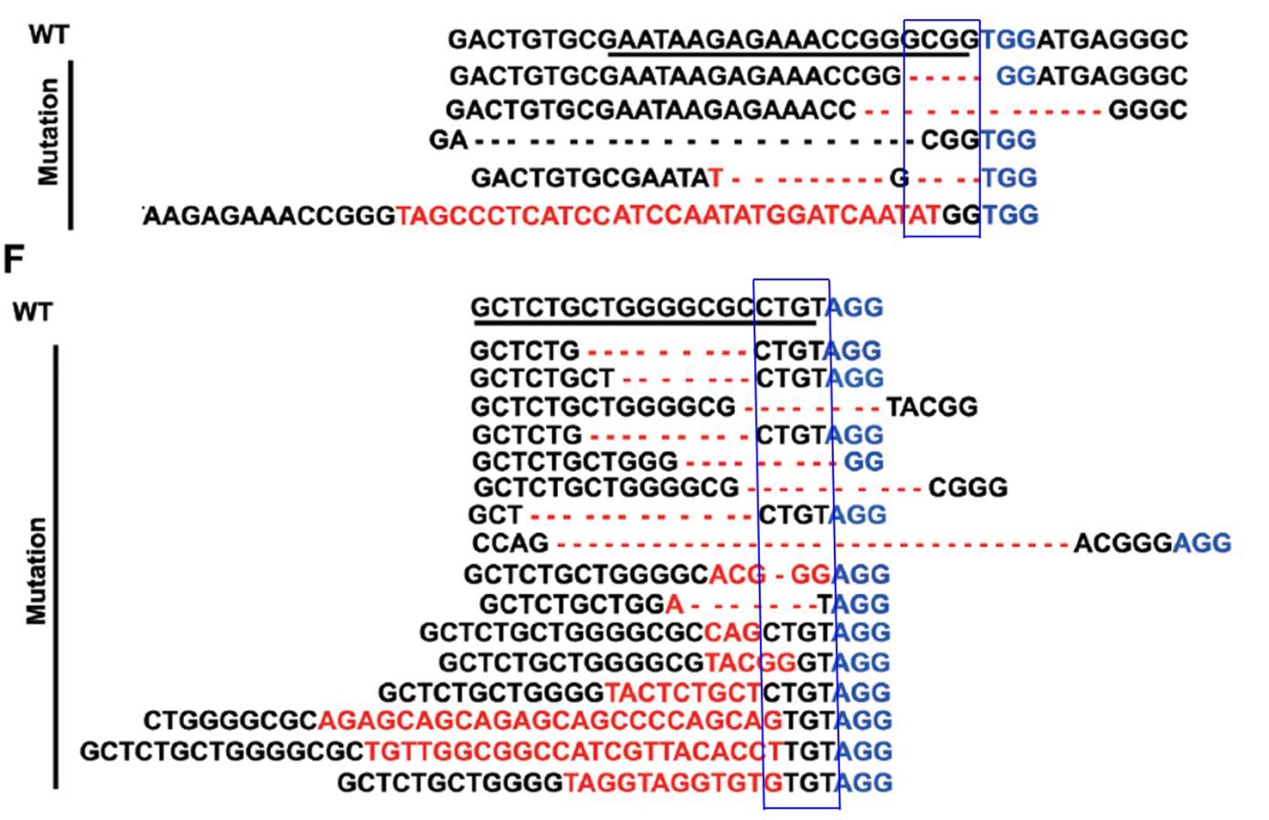
**

Mutation sequences: 20

Mutations including 4 bases: 8

Mutations including 3 bases: 1

Mutations including 2 bases: 2

Mutations including 1 base: 4

**
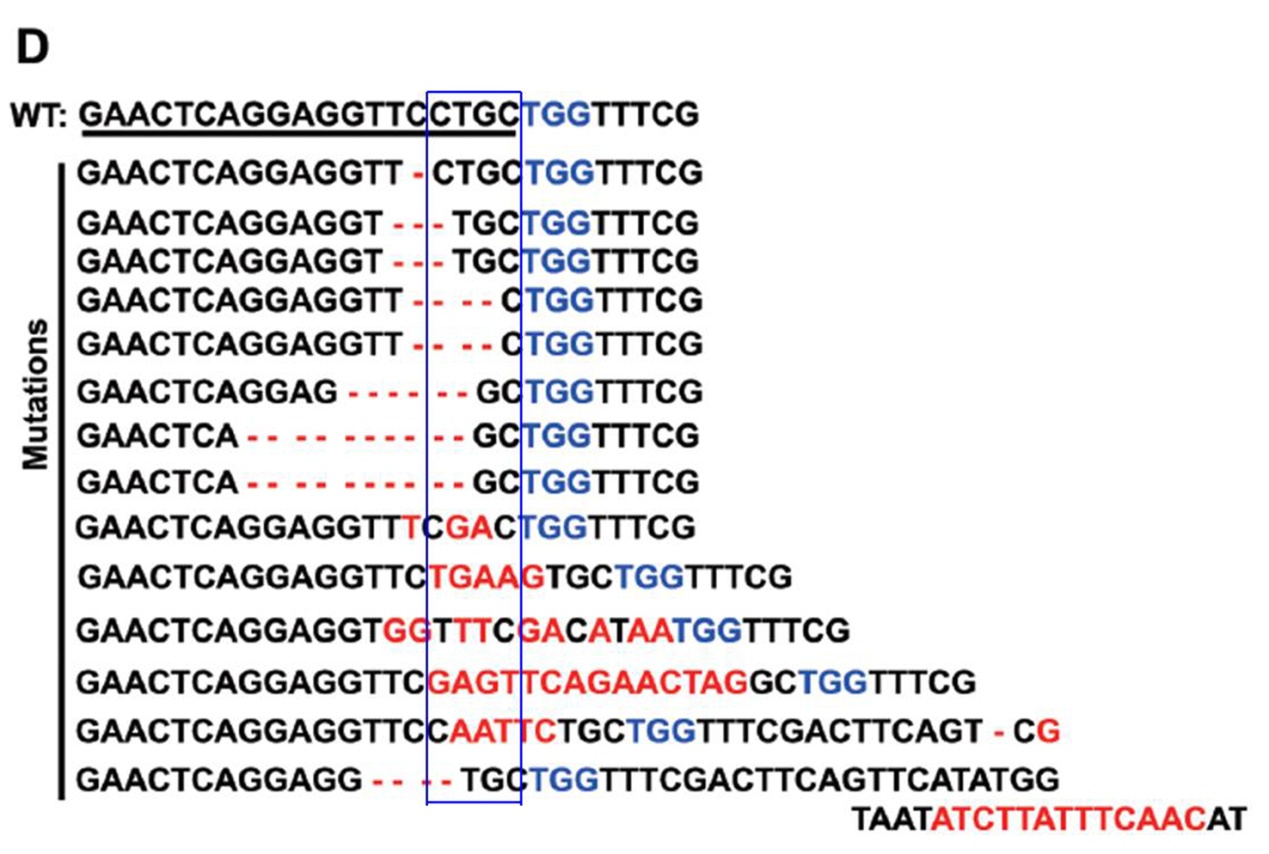
**

Mutation sequences: 14

Mutations including 4 bases: 2

Mutations including 3 bases: 3

Mutations including 2 bases: 5

Mutations including 1 base: 3

**Reference 6:** DiCarlo JE, Norville JE, Mali P, *et al*. (2013) Genome engineering in Saccharomyces cerevisiae using CRISPR-Cas systems. Nucleic Acids Research, 1–8.

**
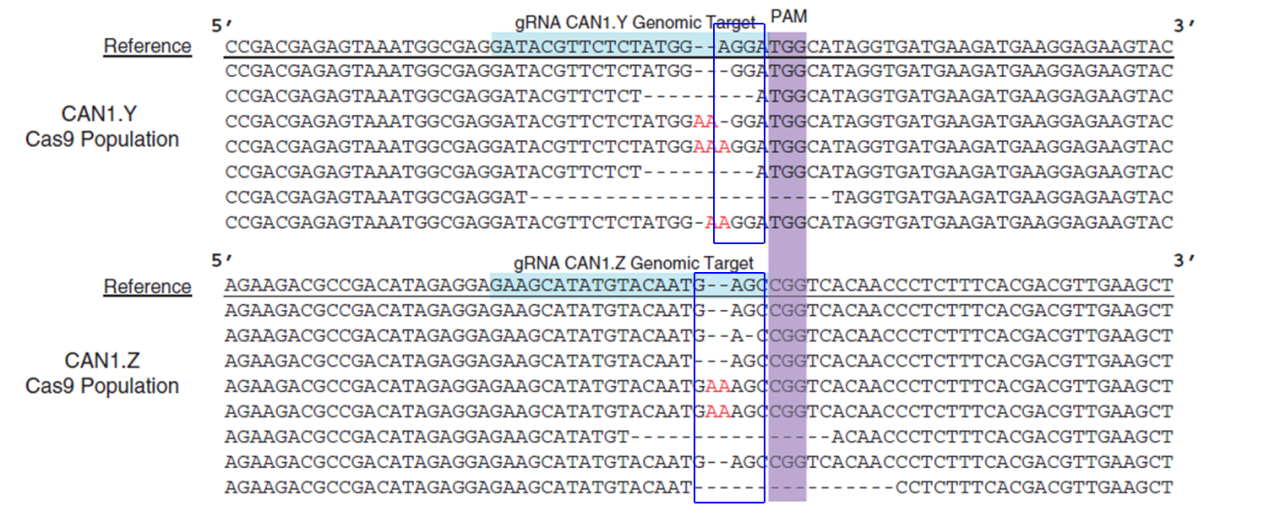
**

Mutation sequences: 15

Mutations including 4 bases: 3

Mutations including 3 bases: 2

Mutations including 2 bases: 0

Mutations including 1 base: 8

**Reference 7:** Bassett AR, Tibbit C, Ponting CP, *et al*. (2013)Highly Efficient Targeted Mutagenesis of Drosophila with the CRISPR/Cas9 System. Cell Reports, 4: 220–228.


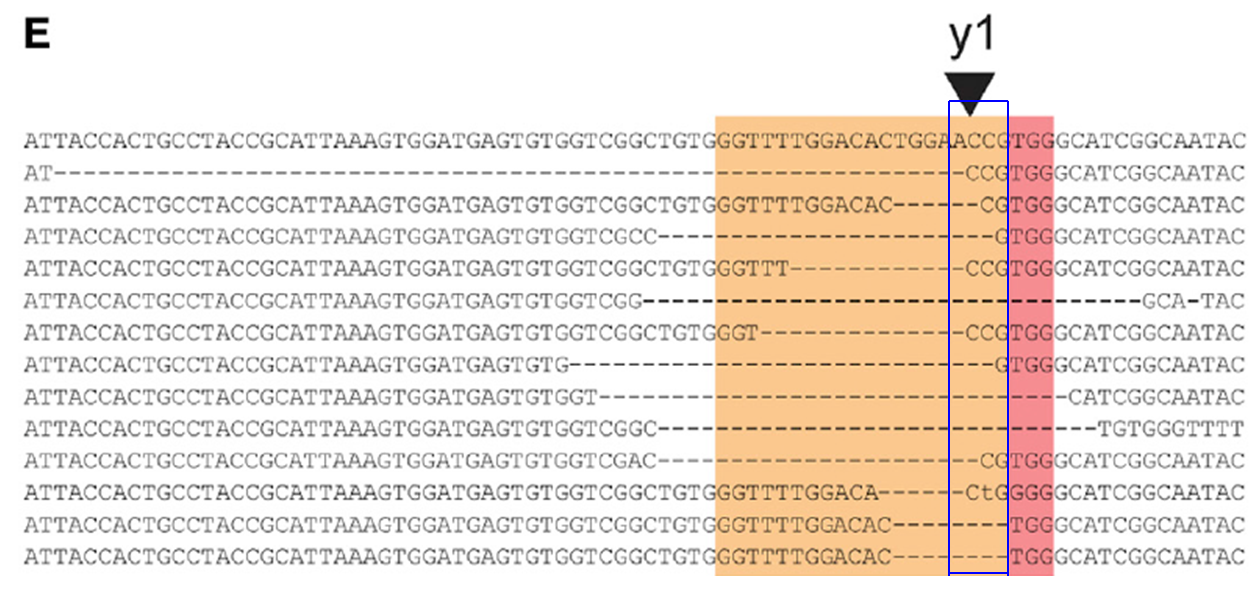


Mutation sequences: 15

Mutations including 4 bases: 7

Mutations including 3 bases: 2

Mutations including 2 bases: 3

Mutations including 1 base: 3


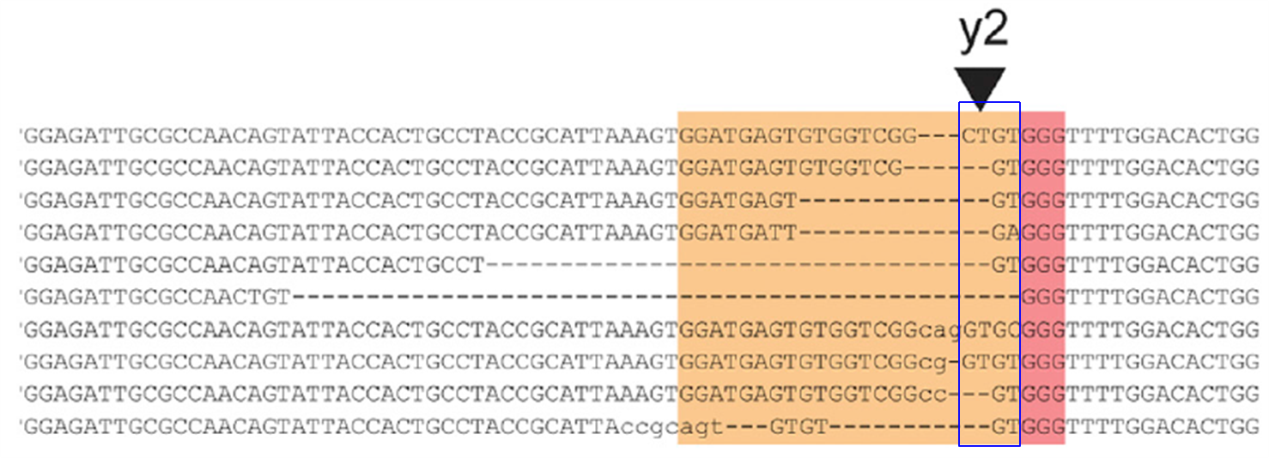


Mutation sequences: 9

Mutations including 4 bases: 1

Mutations including 3 bases: 0

Mutations including 2 bases: 6

Mutations including 1 base: 0


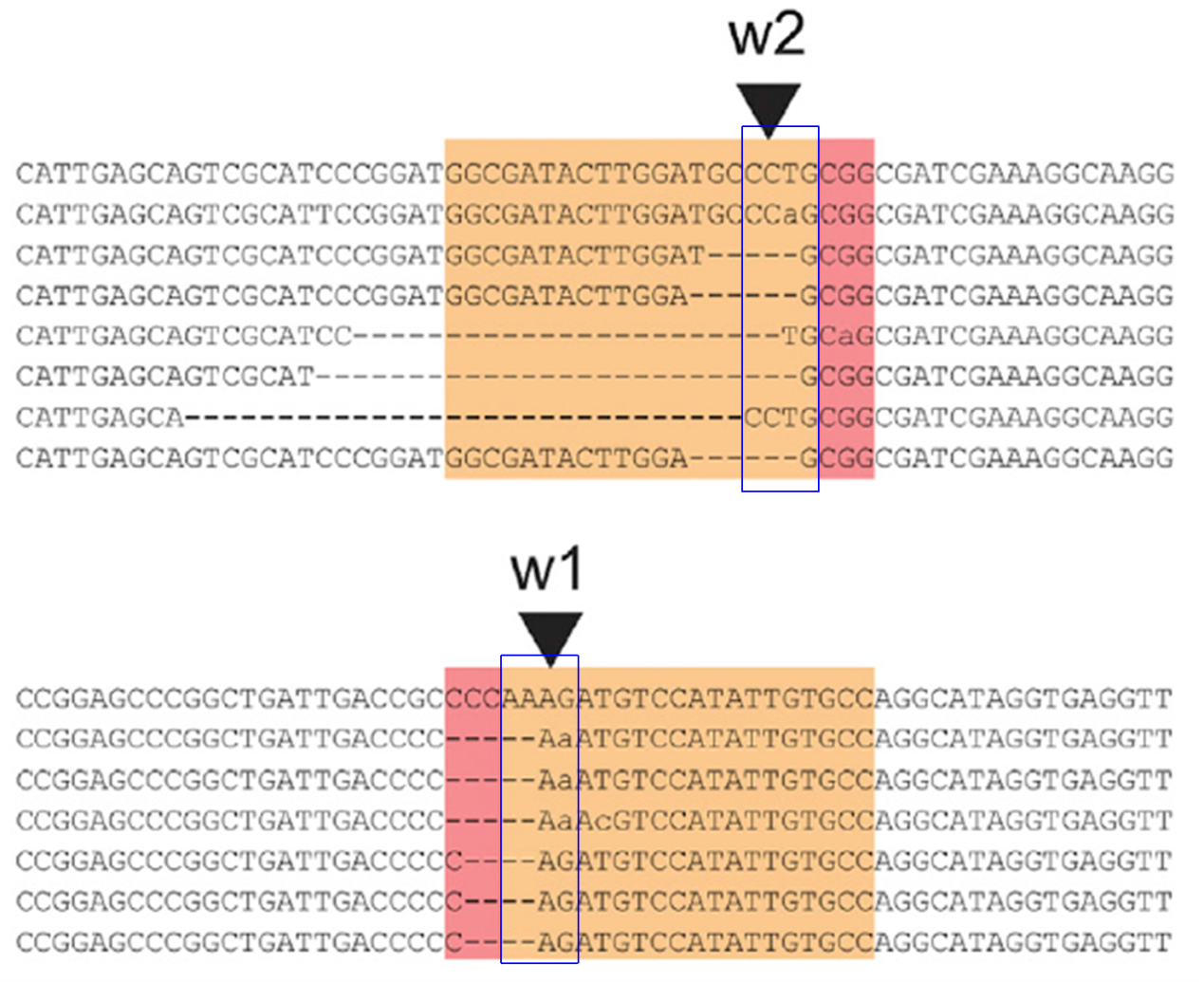


Mutation sequences: 13

Mutations including 4 bases: 0

Mutations including 3 bases: 7

Mutations including 2 bases: 4

Mutations including 1 base: 1

**Reference 8:** Friedland AE, Tzur YB, Esvelt KM, *et al*. Heritable genome editing in *C. elegans* via a CRISPR-Cas9 system. Nature Methods, 10(8): 741–743.


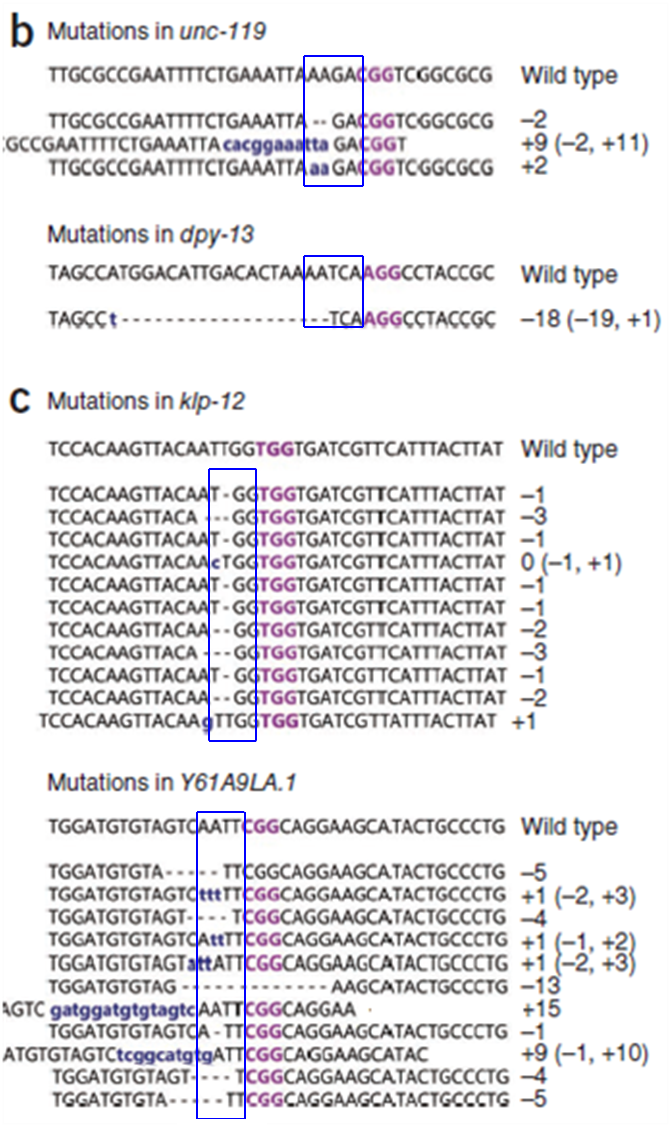


Mutation sequences: 25

Mutations including 4 bases: 1

Mutations including 3 bases: 2

Mutations including 2 bases: 11

Mutations including 1 base: 10

**Reference 9:** Yu Z, Ren M, Wang Z, *et al*. (2013) Highly Efficient Genome Modifications Mediated by CRISPR/Cas9 in Drosophila. Genetics, 195: 289–291.

**Supplementary Materials**


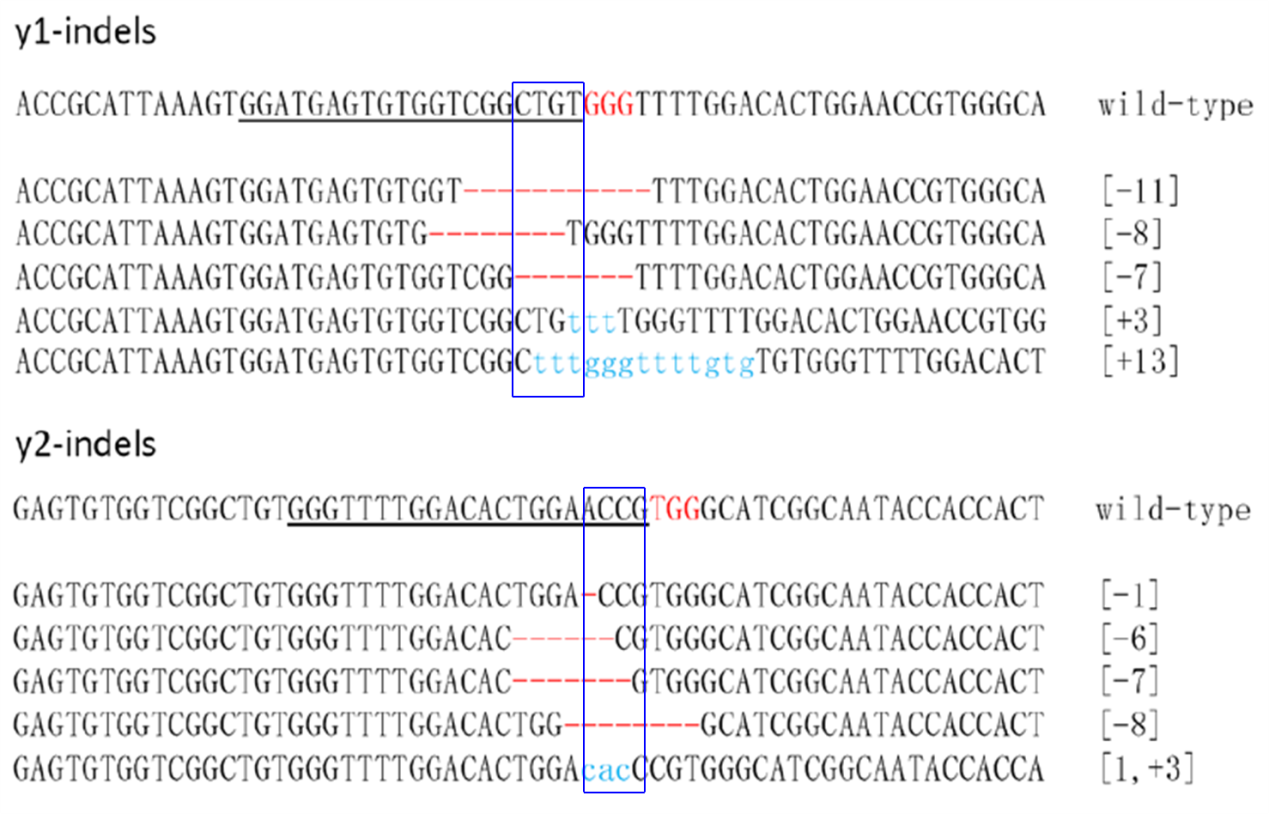


Mutation sequences: 10

Mutations including 4 bases: 3

Mutations including 3 bases: 4

Mutations including 2 bases: 1

Mutations including 1 base: 2


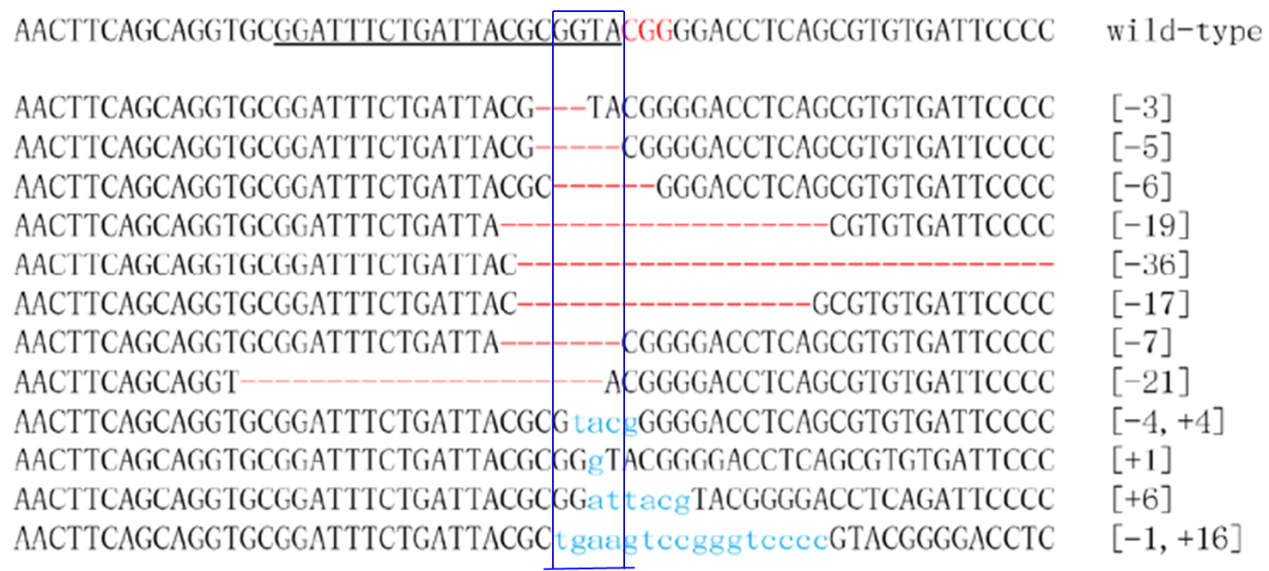


Mutation sequences: 12

Mutations including 4 bases: 7

Mutations including 3 bases: 2

Mutations including 2 bases: 2

Mutations including 1 base: 1


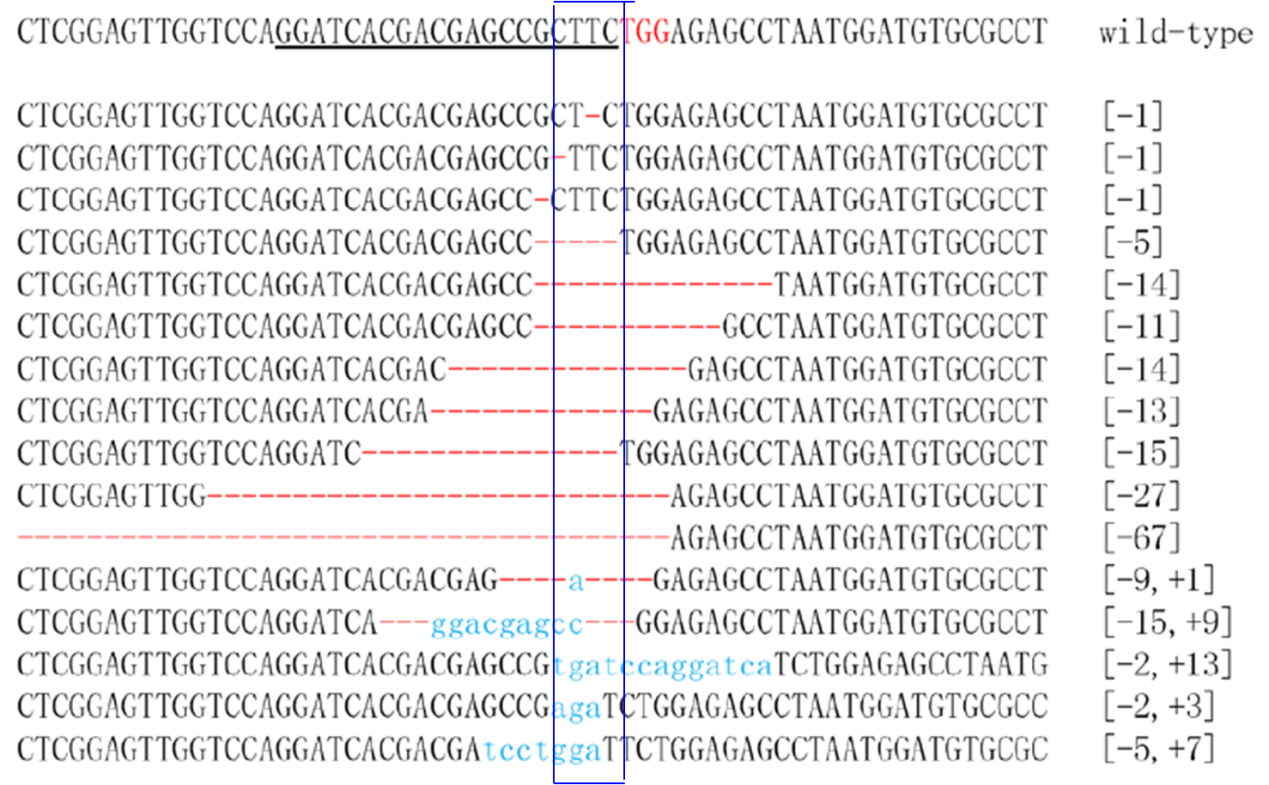


Mutation sequences: 16

Mutations including 4 bases: 11

Mutations including 3 bases: 2

Mutations including 2 bases: 0

Mutations including 1 base: 2


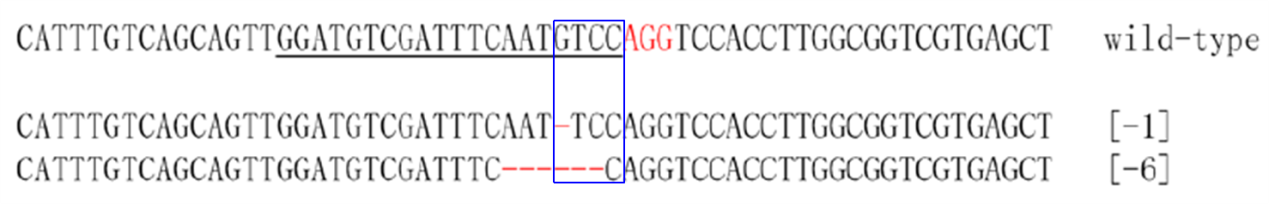


Mutation sequences: 2

Mutations including 4 bases: 0

Mutations including 3 bases: 1

Mutations including 2 bases: 0

Mutations including 1 base: 1


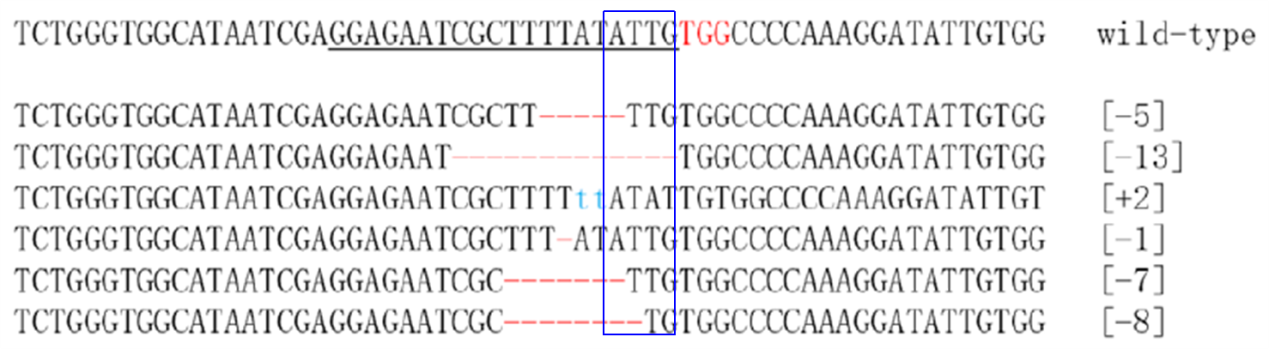


Mutation sequences: 6

Mutations including 4 bases: 1

Mutations including 3 bases: 0

Mutations including 2 bases: 1

Mutations including 1 base: 2


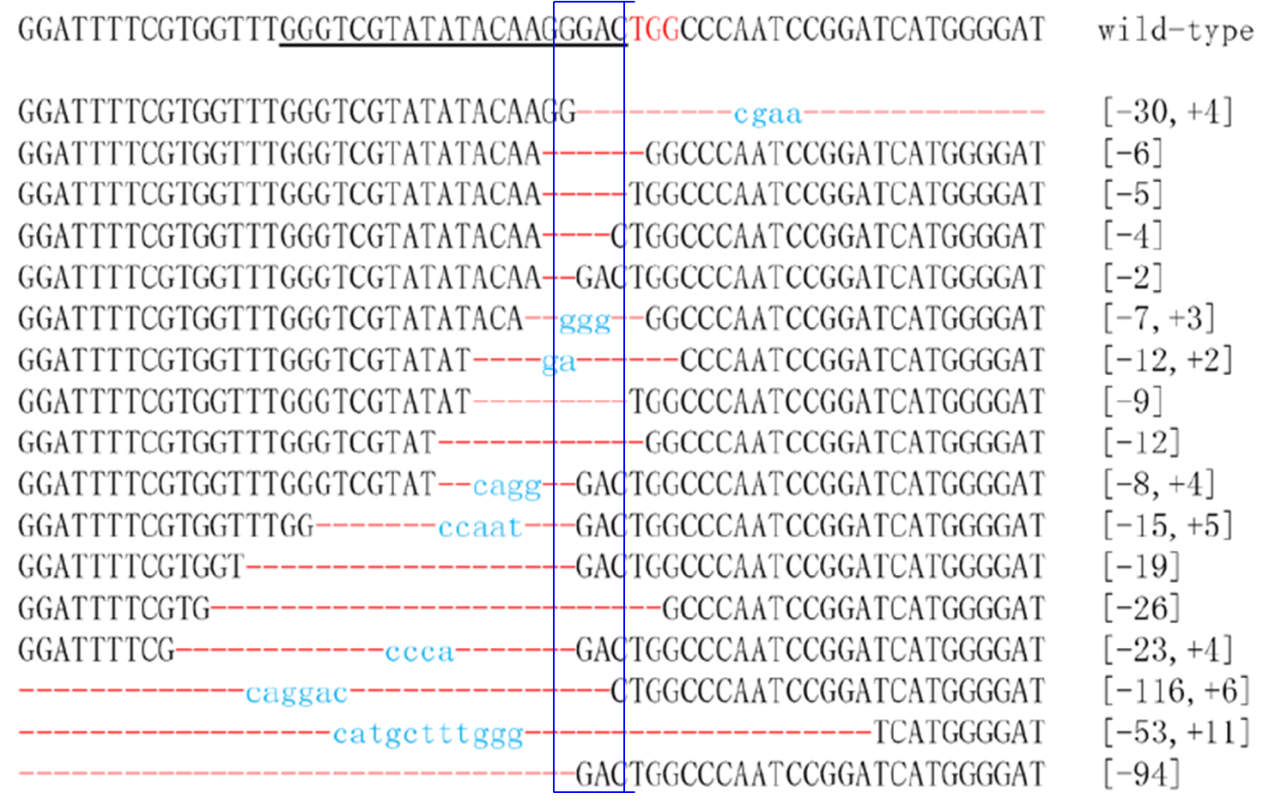


Mutation sequences: 17

Mutations including 4 bases: 8

Mutations including 3 bases: 3

Mutations including 2 bases: 0

Mutations including 1 base: 6


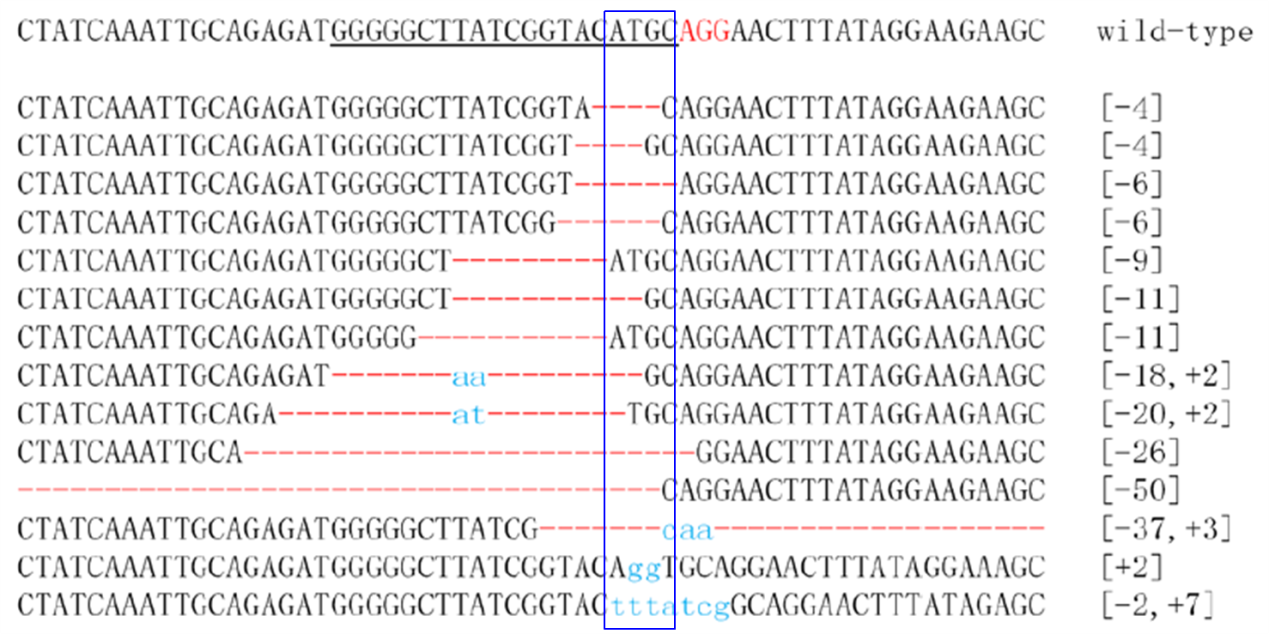


Mutation sequences: 14

Mutations including 4 bases: 4

Mutations including 3 bases: 3

Mutations 2 bases: 4

Mutations 1 base: 1

**Table 1**. Cas9 induced mutant sequence statistics

|  | Mutation type | Mutant sequence number | ratio |
| --- | --- | --- | --- |
| Mutational hot spot regions (MHS): the 4 base pairs upstream of the PAMs | Mutations (4 bases) | 166 | 42.67% |
|  | Mutations (3 bases) | 68 | 17.48% |
|  | Mutations (2 bases) | 58 | 14.91% |
|  | Mutations (1 bases) | 79 | 20.31% |
|  | All mutants at MHS | 371 | 95.37% |
|  | Total mutant sequences | 389 |  |
